# A new phylogeny-based tribal classification of subfamily Detarioideae, an early branching clade of florally diverse tropical arborescent legumes

**DOI:** 10.1038/s41598-018-24687-3

**Published:** 2018-05-02

**Authors:** Manuel de la Estrella, Félix Forest, Bente Klitgård, Gwilym P. Lewis, Barbara A. Mackinder, Luciano P. de Queiroz, Jan J. Wieringa, Anne Bruneau

**Affiliations:** 1 Comparative Plant and Fungal Biology Department, Royal Botanic Gardens, Kew, Richmond, TW9 3DS UK; 2 Departamento de Botánica, Ecología y Fisiología Vegetal, Facultad de Ciencias, Campus de Rabanales, Universidad de Córdoba, 14071 Córdoba, Spain; 3 Department for Identification and Naming, Royal Botanic Gardens, Kew, Richmond, TW9 3AE UK; 4 Tropical Diversity, Royal Botanic Garden Edinburgh, 20ª Inverleith Row, EH3 5LR Edinburgh, UK; 5 Departamento de Ciências Biológicas, Universidade Estadual de Feira de Santana, Av. Transnordestina s.n., Novo Horizonte, 44036-900 Feira de Santana, Bahia Brazil; 6 Naturalis Biodiversity Centre, National Herbarium of the Netherlands, Darwinweg 2, 2333 CR Leiden, The Netherlands; 7 Institut de recherche en biologie végétale and Département de Sciences biologiques, Université de Montréal, 4101 Sherbrooke est, Montréal, H1X 2B2 Canada

## Abstract

Detarioideae (81 genera, c. 760 species) is one of the six Leguminosae subfamilies recently reinstated by the Legume Phylogeny Working Group. This subfamily displays high morphological variability and is one of the early branching clades in the evolution of legumes. Using previously published and newly generated sequences from four loci (*matK*-*trnK*, *rpL16*, *trnG-trnG2G* and ITS), we develop a new densely sampled phylogeny to assess generic relationships and tribal delimitations within Detarioideae. The ITS phylogenetic trees are poorly resolved, but the plastid data recover several strongly supported clades, which also are supported in a concatenated plastid + ITS sequence analysis. We propose a new phylogeny-based tribal classification for Detarioideae that includes six tribes: re-circumscribed Detarieae and Amherstieae, and the four new tribes Afzelieae, Barnebydendreae, Saraceae and Schotieae. An identification key and descriptions for each of the tribes are also provided.

## Introduction

The Detarioideae is a monophyletic group of legumes (Leguminosae or Fabaceae) with an astonishing morphological diversity that comprises c. 760 species in 81 genera distributed across the tropical regions of the world^1–4^. This lineage is one of the first branches in the legume phylogeny and it was recently reinstated as subfamily Detarioideae Burmeist. in the new classification of the family proposed by the Legume Phylogeny Working Group^3^, which recognizes six subfamilies.

Despite its pantropical distribution, the majority of the detarioid generic and species diversity occurs in Africa and Madagascar (58% of genera and c. 330 spp.), followed by Central and South America (20% of genera and c. 247 spp.), and Asia (12% of genera and c. 124 spp.)^2^. The Detarioideae include many ecologically important tree species in West Central African lowland evergreen rainforests^5–7^, and in some forest types trees of this subfamily are the dominant species (e.g., *Brachystegia* woodland, monodominant *Gilbertiodendron* forests or *Microberlina* dominated groves^6,8^). Some Detarioideae species are also ecologically important components in lowland wet forests of the Neotropics (e.g., *Brownea*, *Copaifera*, *Macrolobium*, and *Peltogyne* species^9–11^). In contrast, in Asian tropical dipterocarp-dominated rainforests, although present, Detarioideae represent a modest fraction of the species abundance and diversity^12,13^. Plants of this subfamily provide timber (e.g. *Aphanocalyx*, *Berlinia*, *Didelotia*, *Hymenaea, Peltogyne* and *Tetraberlinia*), some of which are highly valuable (e.g., species of *Guibourtia*), several species are the source of useful resins (e.g. *Copaifera*, *Hymenaea*), and *Tamarindus* is used as a condiment for cooking^5,14,15^. Some species are also part of cultural heritage, used for rituals and medicine or seen as holy trees (e.g. several species of *Brownea*^16^ and *Copaifera religiosa*^17^).

**Figure 1:**
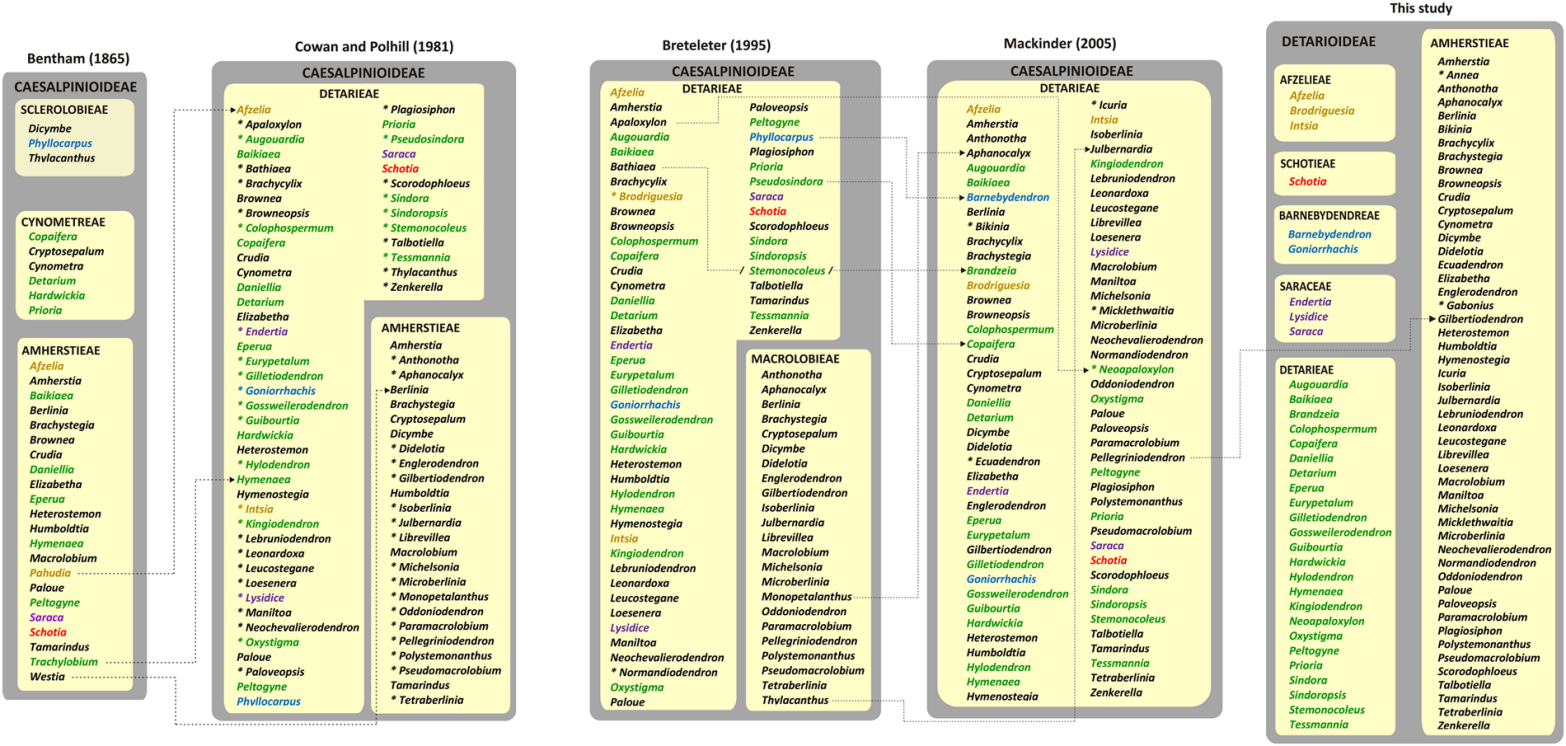
Generic composition of the Detarioideae based on four prior taxonomic treatments and the present study. Dotted lines indicate changes in genus circumscription or transfer between tribes. Genera described after the previous treatment are indicated by an asterisk.

Since the mid-1800, the generic content of Detarioideae has remained relatively stable, but the higher level subdivision, into one or two tribes or subtribes, has fluctuated considerably (Fig. 1). Lee and Langenheim^18^ provided an historical review of the tribal classification of detarioid legumes, starting with the publication of the tribe Detarieae in de Candolle’s *Prodromus*^19^). Bentham^20,21^ established seven tribes within his 2^nd^ legume suborder, Caesalpinieae. Two of these tribes, Amherstieae and Cynometreae, included genera ascribed to tribe Detarieae (sensu Mackinder^2^). The tribe Sclerolobieae was later merged with tribe Cynometreae^22,23^. Based on a detailed study of seedlings of African genera, Léonard^24^ classified the detarioid legumes in two tribes (Cynometreae and Amherstieae), which were later slightly modified by Heywood^25^ who gave priority to the name Detarieae over Cynometreae. These tribal circumscriptions were largely followed by Cowan and Polhill^26,27^. Breteler^28^ adopted a new tribal classification for the Detarieae-Amherstieae association based on bracteole aestivation, whether valvate or imbricate, and recognized two tribes: Detarieae (including some genera transferred from the Amherstieae) and Macrolobieae Breteler (Fig. 1). However, molecular studies subsequently showed that the Macrolobieae is nested with genera previously recognized as part of Amherstieae^29–31^. In the *Phytochemical Dictionary of the Leguminosae*, Polhill^32^ accepted a single tribe Detarieae *s.l*., and this was followed by Mackinder^2^ and subsequent taxonomic treatments.

Phylogenetic studies have demonstrated that no previous tribal circumscriptions are supported as monophyletic, but several well-supported clades have been resolved within Detarioideae since the first comprehensive molecular studies attempted to resolve relationships in the group^29,31^. These include the Prioria, Brownea and Amherstieae clades. Subsequent studies have focused on specific clades. Wieringa and Gervais^33^ studied the “babijt” clade including the *Aphanochalyx*-*Bikinia*-*Tetraberlinia* group, which also received support from a chemical analysis^34^. Fougère-Danezan *et al*.^35–37^ studied the Detarieae in which they recognised the “resin-producing Detarieae”, a group that comprises the Detarieae *s.s*. and the Prioria clade, and which produces bicyclic diterpenes^36^. Other phylogenetic studies have focused on subsets of Detarioideae genera (e.g.,^5,10,15,35,38–42^). More recently Estrella *et al*.^43^ studied the biogeographic origin of the subfamily proposing a probable *terra firme* African origin in the Palaeocene with subsequent and frequent early dispersals to South America and Asia.

The recently published subfamily framework for legumes^3^ highlighted the need for new classifications at the supra-generic level of some of the six recognised subfamilies. Phylogeny-based classifications of taxonomically complex, ecologically diverse and morphologically heterogeneous clades such as the Detarioideae are essential to pave the way for further taxonomic studies of genera and groups of genera, as well for tracking the course of morphological evolution, speciation and extinction patterns, and biome shifts. The objective of the present study is to produce a new tribal classification that reflects current knowledge of phylogenetic relationships in Detarioideae, supported by a near complete generic level sampling and a representative species level sampling.

## Material and Methods

### Taxon sampling

A total of 501 accessions, representing 280 species of Detarioideae from 73 of the 81 genera were sampled. Additionally, two genera of subfamily Cercidoideae and one each of Duparquetioideae and Caesalpinioideae were sampled as outgroups. This is the broadest sampling of Detarioideae species assembled to date for phylogenetic analysis (Supplementary Appendix I provides voucher information and GenBank accession numbers). Samples collected in the field were preserved in silica gel, and other samples were obtained from dried herbarium specimens. We generated most of the sequences (including 475 sequences newly released for this study), and the sampling was completed with additional sequences produced by our research group in previous studies^30,36,39,40,44^ which were downloaded from GenBank (http://www.ncbi.nlm.nih.gov/genbank/) to complete the taxon and gene sampling. To avoid the effects of missing data no sample was included that had fewer than two loci sequenced, and for this reason six genera that have been included in other studies (*Brachycylix*, *Lebruniodendron*, *Micklewaitia*, *Michelsonia, Neoapaloxylon, Paloveopsis*) are not included in our analyses. We were not able to obtain material of *Leucostegane* and *Pseudomacrolobium* for sequencing.

### Molecular methods

DNA extraction of herbarium and silica gel dried material was done using a modified protocol from Ky *et al*.^45^ rescaled for a total 3 mL of nucleic extraction buffer (15 mM Tris, 2 mM EDTA, 80 mm KCl, 20 mM NaCl, 2% β-mercaptoethanol, PPVP 2%, 0.5% Trixon-X100) and the pellet was recovered in 2 ml of lysis buffer pH 8 (0.1 M Tris, 0.02 M EDTA, 1.25 M NaCl, MATAB 4%).

Three plastid (*matK*-*trnK*, *rpL16* and *trnG*-*trnG2G*) regions and the nuclear ribosomal internal transcribed spacers (ITS/5.8 S) were amplified and sequenced. The PCR amplification mix in reaction volumes of 50 μL contained 4 units of Taq DNA polymerase, 1× Taq DNA polymerase buffer with 1.5 mmol MgCl_2_ (New England Biolabs, Pickering, Ontario, Canada), 200 μmol/L of each dNTP (Fermentas, Burlington, Ontario, Canada), 3 μmol/L of each primer, and 50–100 ng of genomic DNA. For recalcitrant samples, BSA (0.1 μg/μL, New England BioLabs, Ipswich, Mass.), Tween 20 (0.03%, J-T. Baker, Phillipsburg, New Jersey, USA), and pure DMSO (4%, Fisher Scientific, Ottawa, Ontario, Canada) were added to the mix.

For samples that were difficult to amplify, we also used a nested PCR procedure described in Gagnon *et al*.^46^. For the most problematic samples, including those with large mononucleotide repeats, we used a PCR protocol with Phusion Hot Start II High-Fidelity DNA polymerase (Thermo Scientific, Waltham, Massachusetts, U.S.A.), which is more accurate and yields longer and higher-quality mononucleotide sequence reads^47^.

For the ITS/5.8 S region, amplifications were performed with the “AB101” and “AB102” primers^48,49^; conditions for the amplification follow Estrella *et al*.^40^. The *matK* gene and the flanking 3′ intron region were amplified in one fragment using the primers trnK685F and trnK2Rdet^30^ and the internal primers described in that study were used to sequence the most difficult samples. For *trnG-trnG2G* and *rpL16* we used the primers and amplification conditions from Shaw *et al*.^50^, but because *rpL16* was difficult to sequence due to a large adenine repeat, we designed a specific internal primer that we used for sequencing (FX1: 5′-TGGATTATGAGTTGTGAAGC-3′). Sequencing was performed with Big Dye Terminator 3.1 chemistry on an ABI 3730xl DNA Analyzer (Applied Biosystems, Carlsbad, California, USA) at the Genome Quebec facilities (Montreal, Canada).

Sequences were assembled and edited with Geneious 4.8.5 (Biomatters Ltd., http://www.geneious.com). All sequences were subjected to a Blast search^51^ and eliminated if they did not correspond to Leguminosae sequences in GenBank. The *matK-trnK* matrix included 478 sequences from different accessions, the *trnG-trnG2G* matrix included 446 sequences, the *rpL16* included 473 sequences and the ITS/5.8 S matrix included 462 sequences.

### Phylogenetic analyses

Sequence alignment was performed using MAFFT^52^ for the plastid markers and SATé^53–55^ for ITS. We configured the SATé analysis following the approach described in Callahan and McPeek^56^ which initially estimates an alignment and tree with MAFFT^52^ and FASTTREE^57^, decomposes the estimated tree using the longest-edge strategy into subsets no larger than 50% of the tips, aligns each subset with PRANK^58^, merges the PRANK sub-alignments with MUSCLE, estimates a new tree from the merged alignment using RAxML^59^ under a GTRGAMMA model, and repeats this cycle of steps for 10 iterations. Finally, ambiguous sites were removed using Gblocks^60,61^, allowing gap positions under stringent parameter settings. The ITS alignment from the last iteration of the SATé + Gblocks and the plastid alignments were inspected and manually edited using Geneious 4.8.5 (Biomatters Ltd., http://www.geneious.com). The aligned *matK*-*trnK* matrix had a total length of 1941 base pairs (bp), the *trnG*-*trnG2G* had a total length of 1102 bp, the *rpL*16 a total length of 1855 bp, and the *ITS* was 1533 bp in length.

Two matrices (ITS and combined plastid) were analysed separately for exploratory purposes, and a concatenated plastid + nuclear matrix of all data containing only 7% of missing sequences was analysed using Maximum likelihood and Bayesian approaches to generate the phylogenetic trees. Maximum likelihood analyses were carried out using RAxML v.8.0.0^62^, on the CIPRES gateway v.3.3^63^. The analyses were conducted using the GTRGAMMA model. Branch support was assessed using the nonparametric bootstrap procedure, with 1000 replicates. jModelTest v.2^64^ was used to estimate the best evolutionary model for each DNA locus separately. Based on the Akaike information criterion, the best models identified were GTR + I + G for ITS/5.8 S, TVM + G for *matK/trnK* and *rpL16*, and TPM1 uf + G for *trnG-trnG2G*. Bayesian analyses were conducted in MrBayes v.3.2^65^, but because it is not possible to specify the exact models for the three plastid regions in MrBayes, we used the reversible-jump MCMC option, which allows sampling of different schemes of nucleotide substitution as part of the MCMC run (nst = mixed)^46^. The Bayesian estimation consisted of two independent runs during 50 × 10^6^ generations, sampling trees and parameters every 1000th generation. Each run consisted of four simultaneous Monte Carlo Markov Chains, and four swaps per generation. All sample points prior to reaching stationarity of the chains were discarded (equivalent to discarding the first 10% generations as “burn-in”). Convergence was assessed by comparing majority rule consensus trees from the two analyses and by using Tracer version 1.6^66^ to compare density plots of the estimated parameters and of the likelihoods from the two analyses.

## Results

The nuclear and combined plastid datasets converged individually in the Bayesian analyses, but the concatenated plastid + nuclear matrix did not reach convergence. The ITS analyses alone showed poor resolution (results not shown), and although different options were tried for the ITS alignment, the sequences analysed showed signals of saturation. However, the RAxML ITS + plastid topology generally supports the main clades recovered in the concatenated plastid analysis (Fig. S1).

**Figure 2:**
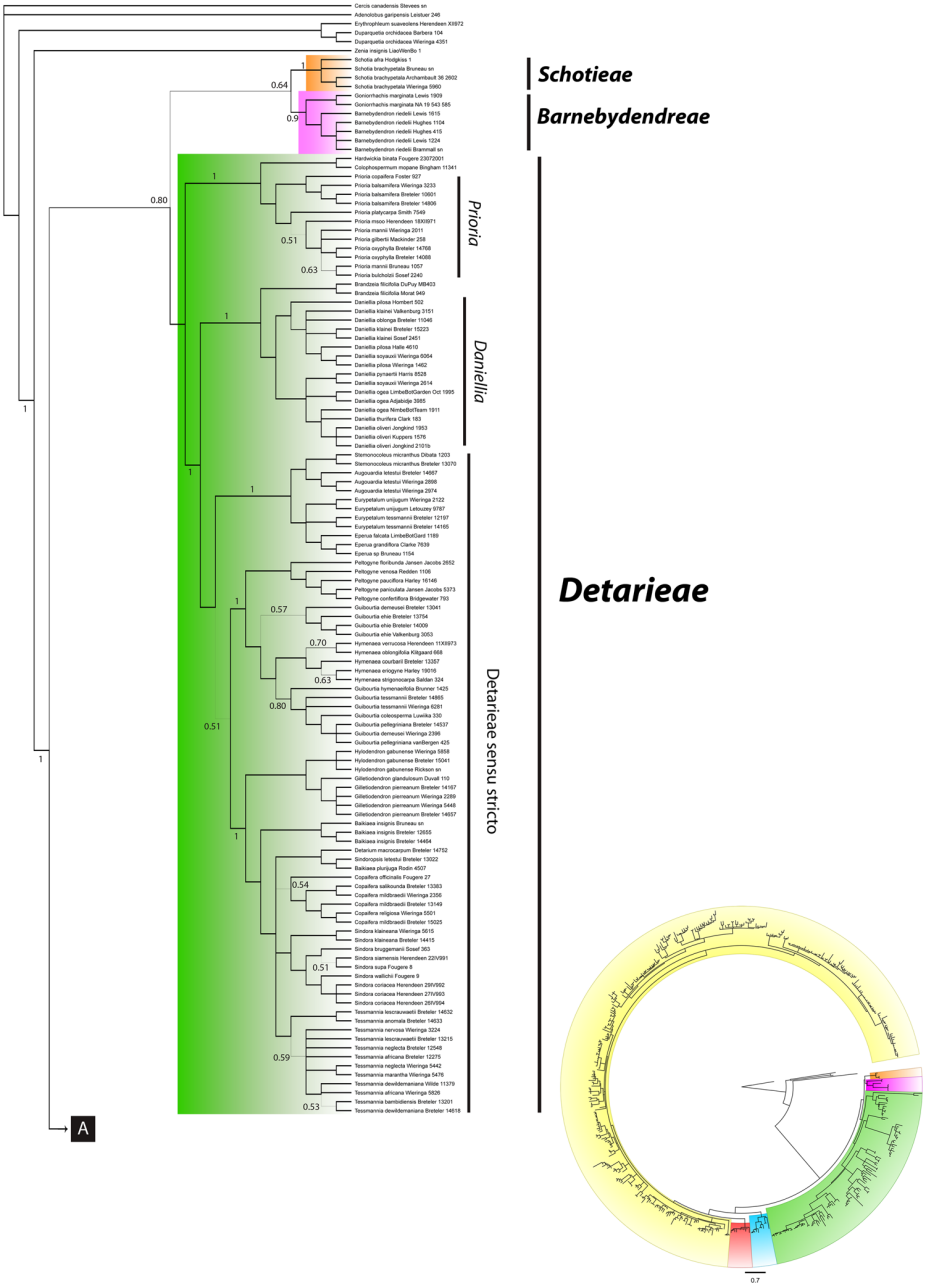
Bayesian majority-rule consensus tree derived from the analysis of the combined three plastid loci (*matK-trnK*, *rpL16*, and *trnG-trnG2G*) for Detarioideae genera. Tree lines width is proportional to the posterior probability (PP), most nodes have PP > 0.9 with wider lines, (principal clades and nodes with PP < 0.9 are printed out). Major clades representing the six recognized tribes are indicated. Collector(s) name(s) and the collection number are indicated after the species name; for further voucher information see Supplementary Appendix I. Names printed in non-italics to allow trees readability.

**Figure 3:**
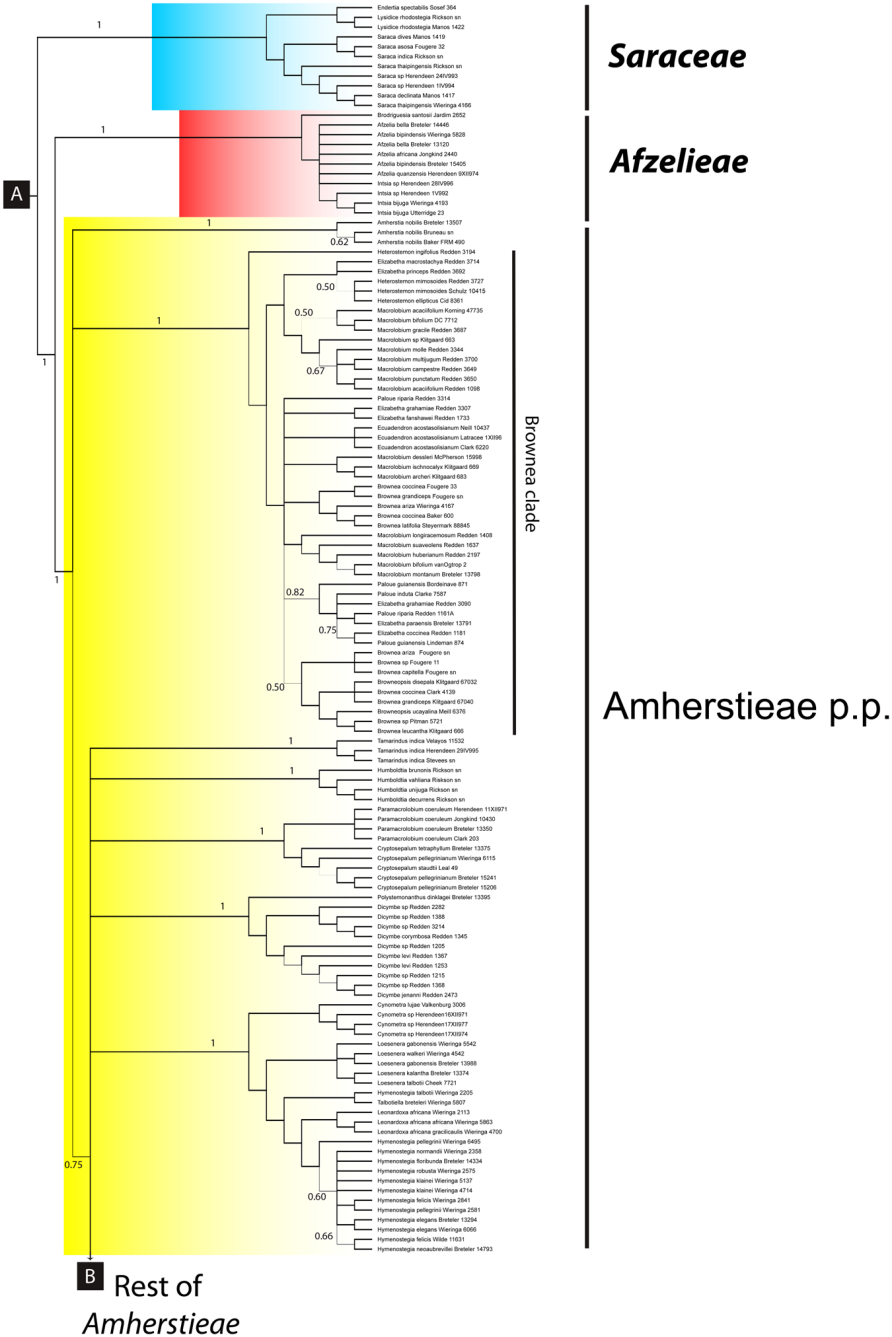
Bayesian majority-rule consensus tree derived from the analysis of the combined three plastid loci (*matK-trnK*, *rpL16*, and *trnG-trnG2G*) for Detarioideae genera. Tree lines width is proportional to the posterior probability (PP), most nodes have PP > 0.9 with wider lines, (principal clades and nodes with PP < 0.9 are printed out). Major clades representing the six recognized tribes are indicated. Collector(s) name(s) and the collection number are indicated after the species name; for further voucher information see Supplementary Appendix I. Names printed in non-italics to allow trees readability.

**Figure 4:**
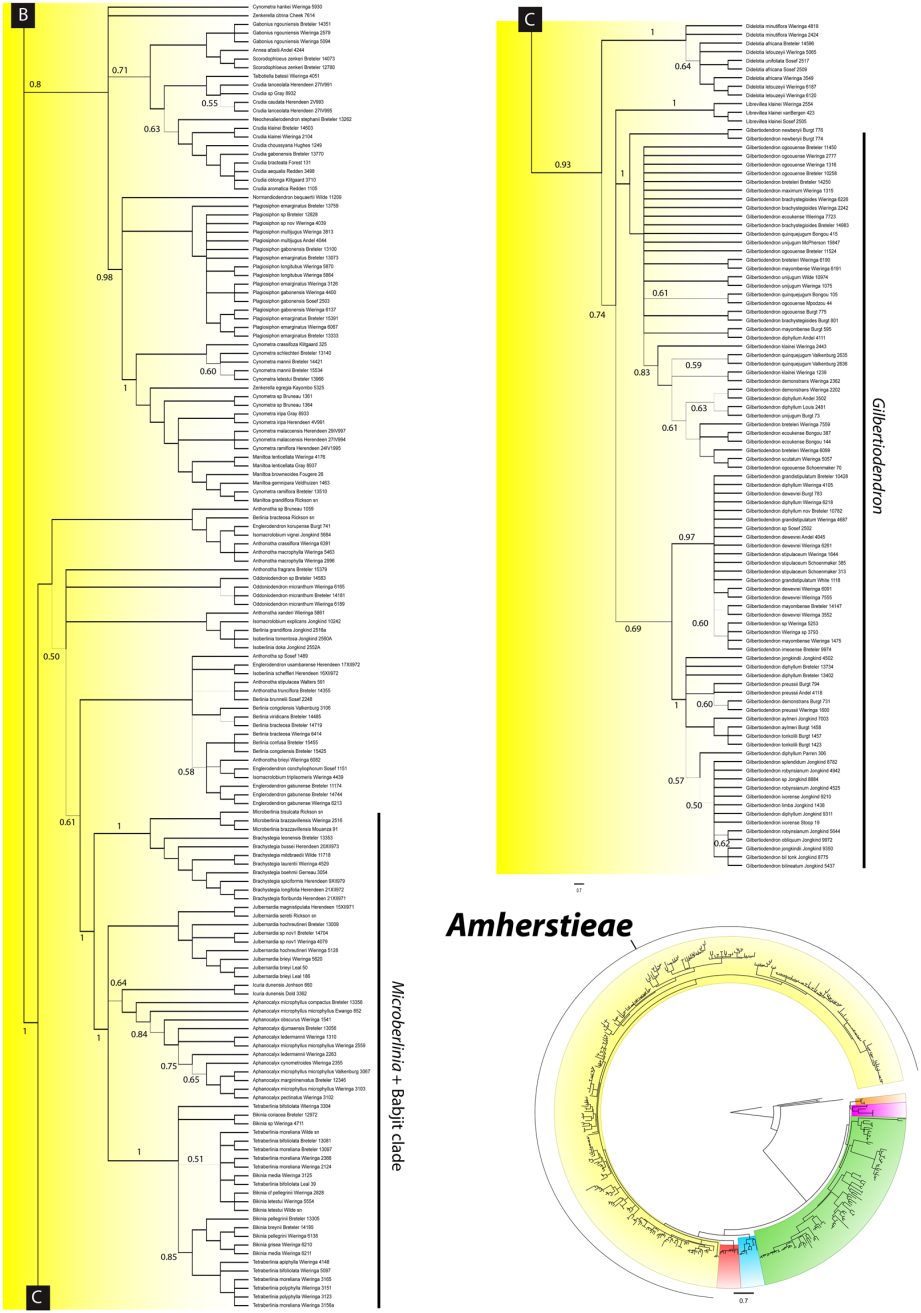
Bayesian majority-rule consensus tree derived from the analysis of the combined three plastid loci (*matK-trnK*, *rpL16*, and *trnG-trnG2G*) for Detarioideae genera. Tree lines width is proportional to the posterior probability (PP), most nodes have PP > 0.9 with wider lines, (principal clades and nodes with PP < 0.9 are printed out). Major clades representing the six recognized tribes are indicated. Collector(s) name(s) and the collection number are indicated after the species name; for further voucher information see Supplementary Appendix I. Names printed in non-italics to allow trees readability.

At the broad level, the analysis of the concatenated plastid markers resolved six major clades. The African genus *Schotia* is resolved as monophyletic [Fig. 2, posterior probability from the Bayesian plastid analysis (PP) = 1; Fig. S1, bootstrap support values from the RaxML cp + ITS analysis (BS) = 100], poorly supported as sister to the American genera *Goniorrhachis* and *Barnebydendron* (Fig. 2). The relationship between these three genera and the resin-producing Detarioideae is only moderately supported in the Bayesian analysis (Fig. 2, PP = 0.8). In the resin-producing Detarioideae, several strongly supported relationships are confirmed, including the monophyly of the genus *Prioria* sensu Breteler^1^, which together with *Colophospermum* and *Hardwickia*, form a clade sister to a Daniellia clade comprised of *Daniellia* plus *Brandzeia* (Fig. 2), sister to another clade formed by the Detarieae *sensu stricto*. In the Detarieae s.s. clade, most genera are supported as monophyletic, except *Guibourtia*, *Copaifera*, and *Baikaea*, and the relationship between *Eperua* and *Eurypetalum* is not well resolved (Fig. 2). The Saraca and Afzelia clades appear as strongly supported successive sister groups to the large Amherstieae clade [Figs 3 and S1, BS = 100%, PP = 1]. The Amherstieae includes most Detarioideae genera, with several moderately to well supported clades recovered. Among these are the Brownea clade that includes seven neotropical genera (PP = 1.0, Fig. 3), a monophyletic group of three African endemic genera, *Didelotia*, *Librevillea* and *Gilbertiodendron* (Fig. 4C, BS = 68%, PP = 1), and a group that includes *Microberlinia*, *Brachystegia* and all of the “Babijt” genera (i.e. *Brachystegia*, *Aphanocalyx*, *Bikinia*, *Icuria*, *Julbernardia* and *Tetraberlinia*) that is only weakly supported as monophyletic (Fig. 4, S1, weak support: PP 0.61, BS < 50%). The monophyly of several genera in the Amherstieae clade is poorly supported (e.g., *Crudia*, *Berlinia*, *Englerodendron*, *Tetraberlinia*) and a few other genera appear to be clearly polyphyletic (i.e., *Cynometra*).

## Discussion

### The new classification

The new Leguminosae classification proposed by the LPWG^3^ follows a traditional Linnaean approach, which as noted by others (e.g.,^67–69^) is compatible and complementary to well-supported clade-based rank-free classifications (e.g. Dalbergioid clade^70^; inverted repeat [IR]-lacking clade,^71^). Because of this new subfamily level classification, certain legume subfamilies require revised classifications. A new classification is particularly needed for the recircumscribed Caesalpinioideae that contains the morphologically distinct mimosoid clade, and where efforts are ongoing to better resolve phylogenetic relationships and to arrive at a new taxonomic treatment^3^. Revising the classification for the pantropical Detarioideae (Detarieae s.l. in Mackinder^2,3^) is also needed. In the past several years a number of studies have been published that aim to understand relationships and evolution in this group (e.g.^1,3–5,10,15,28–31,33,35–41,44,72–76^) and along with the new phylogenetic analysis presented here, we are in a position to present a formal tribal classification of Detarioideae that will provide the necessary framework to better understand the systematics and evolutionary origin of this lineage.

### Phylogenetic evidence

Detarioideae represent an early branching lineage within Leguminosae evolution, estimated at 68–63 Ma^43^, and comprising six strongly supported main clades. These six clades have also been resolved in previous studies; and here we recognize them at the tribe level: Schotieae Estrella, L.P. Queiroz & Bruneau, Barnebydendreae Estrella, L.P. Queiroz & Bruneau, Detarieae DC., Saraceae Estrella, L.P. Queiroz & Bruneau, Afzelieae Estrella, L.P. Queiroz & Bruneau, and Amherstieae Benth.

**Figure 5:**
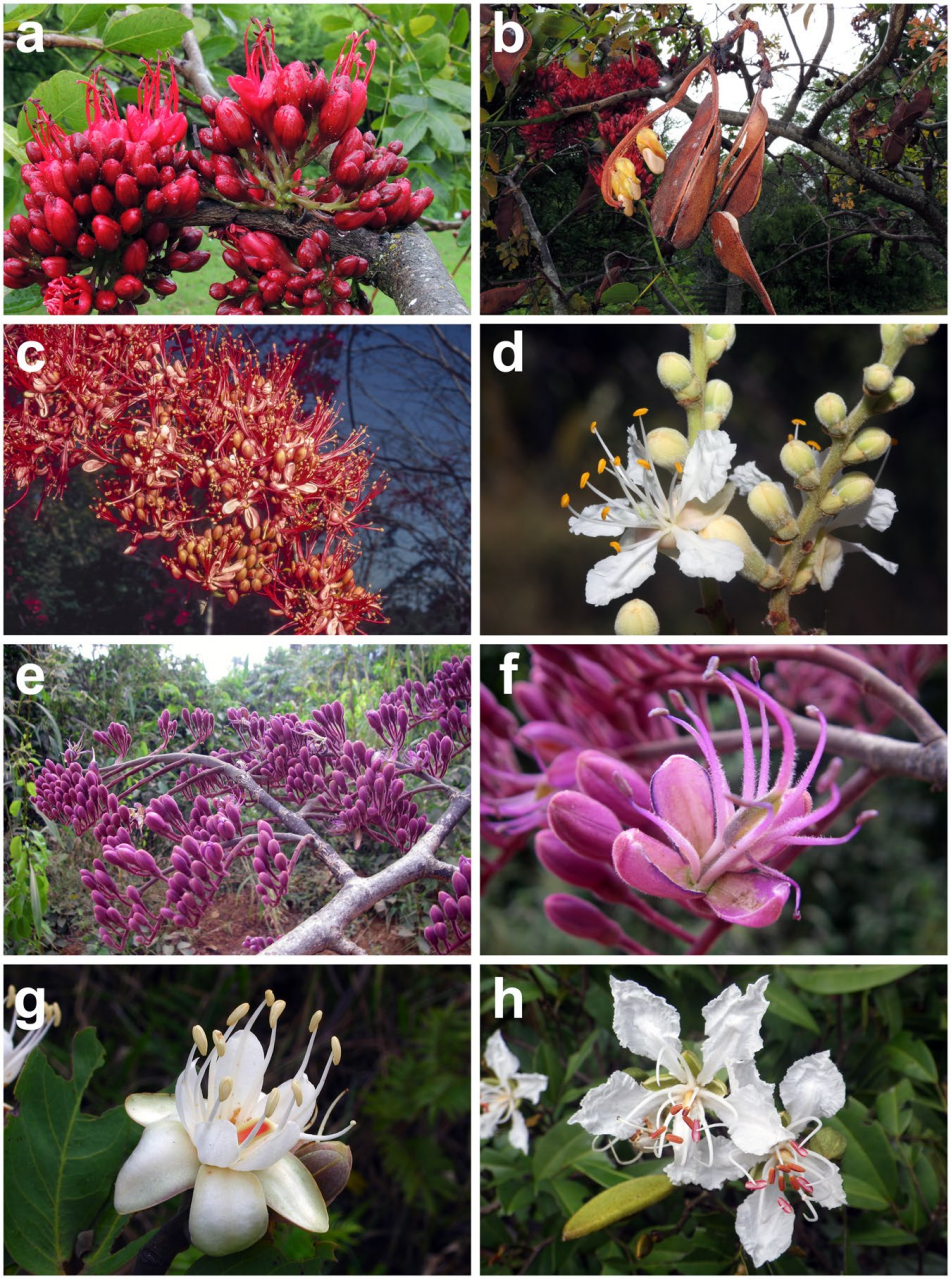
Detarioideae photographs. **(a**,**b)** *Schotia brachypetala* (Schotieae). **(c)** *Barnebydendron riedelii* (Barnebydendreae). **(d)** *Goniorrhachis marginata* (Barnebydendreae). **(e**,**f)** *Daniellia ogea* (Detarieae). **(g)** *Hymenaea stigonocarpa* (Detarieae). **(h)** *Tessmannia baikiaeoides* (Detarieae). — Photos: a & b, E. Moll; c, G.P. Lewis; d, D. Cardoso; e, f & h, X. van der Burgt; g. L. P. de Queiroz.

Three genera, *Schotia*, *Goniorrhachis* and *Barnebydendron*, always appear among the early branching clades within Detarioideae^29–31,43^, and in our analyses these are resolved as sister to the resin-producing Detarioideae, although this relationship is weakly supported (Figs 2, 5). *Schotia* (four species) has been consistently resolved as monophyletic in all analyses (Fig. 2;^29–31,35,36,77^) but its position within the Detarioideae remains unresolved. Depending on the molecular marker or phylogenetic method, it appears as sister to *Goniorrhachis* and *Barnebydendron* (Figs 2, 5), as sister to the resin-producing Detarioideae^36^ or in a polytomy at the base of the subfamily^30^. This unique southern African lineage is thus recognized here as the new monogeneric tribe Schotieae. Morphologically, *Schotia* can be differentiated from most other Detarioideae by its radially symmetrical flowers, with small bracteoles, four upright coloured sepals, five petals some of which can be filamentous, ten mostly free stamens, and a tubular hypanthium^4,78^. The phylogenetic position of *Goniorrhachis* and *Barnebydendron*, two neotropical monospecific genera, also is not fully resolved, however the two genera consistently group together in a highly supported clade^30,35,42^ here recognized under the new tribe Barnebydendreae (Figs 2, 5). As noted by Herendeen *et al*.^42,79^, members now allocated to the Barnebydendreae share the presence of a vein along the margin of the leaflets, a character used by Cowan and Polhill^27^ to discuss subgroups within Detarieae. The two species also share a deep hypanthium^4,80–82^. Although it is possible to argue that these three genera should be included in a single tribe Schotieae, the phylogenetic pattern obtained here and in previous studies^29–31,43^ do not allow us to unequivocally conclude that *Schotia* forms a monophyletic group with *Goniorrhachis* and *Barnebydendron*. This approach with increased division at the tribal level provides a stricter phylogenetic framework for testing evolutionary hypotheses because we do not assume that the two lineages, which morphologically are also very distinct, are necessarily sister clades.

We re-circumscribed tribe Detarieae (Figs 2, 5) as equivalent to the resin-producing clade of previous phylogenetic studies^30,36,43^. This clade was named subtribe Detariinae by Fougère-Danezan *et al*.^35^. This redefined Detarieae is now clearly circumscribed as grouping the 16 genera of Detarieae *s.s*. (*sensu* Fougère-Danezan *et al*.^35^), along with *Colophospermum*, *Hardwickia*, *Prioria*, *Daniellia* and *Brandzeia*. As noted by Fougère-Danezan *et al*.^35,36^ most, but not all, species in this clade produce a characteristic resin composed of various sesquiterpenes and diterpenes^83,84^. A few genera either lack resins or have never been tested for their presence (*Sindoropsis*, *Baikaea*, *Eurypetalum*, *Stemonocoleus*, *Augouardia*, *Hardwickia*^36^;). Few morphological synapomorphies characterise this clade, however, the genera share a combination of characters including: generally caducous stipules, leaves with few leaflets, bracteoles that are often caducous, ten stamens, a strong tendency to apetaly, and most characteristically gland-dotted leaflets (the glands are also often present on the sepals).

Certain generic relationships are now better supported than in previous studies. For example, the monotypic Madagascan genus *Brandzeia*, which occurs in seasonally dry woodlands^2,85^, is resolved as sister to the monophyletic endemic African genus *Daniellia* as also found by Bruneau *et al*.^30^ and Fougere-Danezan *et al*.^35^ but with stronger support in our analyses. *Daniellia* includes species found in both rain forest and savanna biomes^86^. In our analyses, a narrower circumscription of the Prioria clade is strongly supported as monophyletic (Fig. 2). Breteler^1^ subsumed *Gossweilerodendron*, *Kingiodendron* and *Oxystigma* under a broadly defined *Prioria*, a taxonomy that is in accordance with our analyses. Although all the previously recognized genera form monophyletic groups, some are only weakly supported lending support for a more inclusive definition of *Prioria* (Fig. 2), which is what we follow in our tribal classification. Despite the dense taxon sampling presented here, some intergeneric relationships remain unclear. For example, relationships amongst *Tessmannia*, *Sindora*, *Sindoropsis*, *Detarium* or *Copaifera* remain unresolved (Fig. 2). Our study suggests that *Hymenaea* may be nested within a paraphyletic *Guibourtia*, as noted in previous studies^36,43^, and that together these two genera are strongly supported as sister to *Peltogyne*. Fougère-Danezan *et al*.^35^ noted that the three genera have similar bifoliolate leaves with strongly asymmetrical leaflets with a primary vein close to the distal margin of the leaflet and a stipule insertion that is lateral.

**Figure 6:**
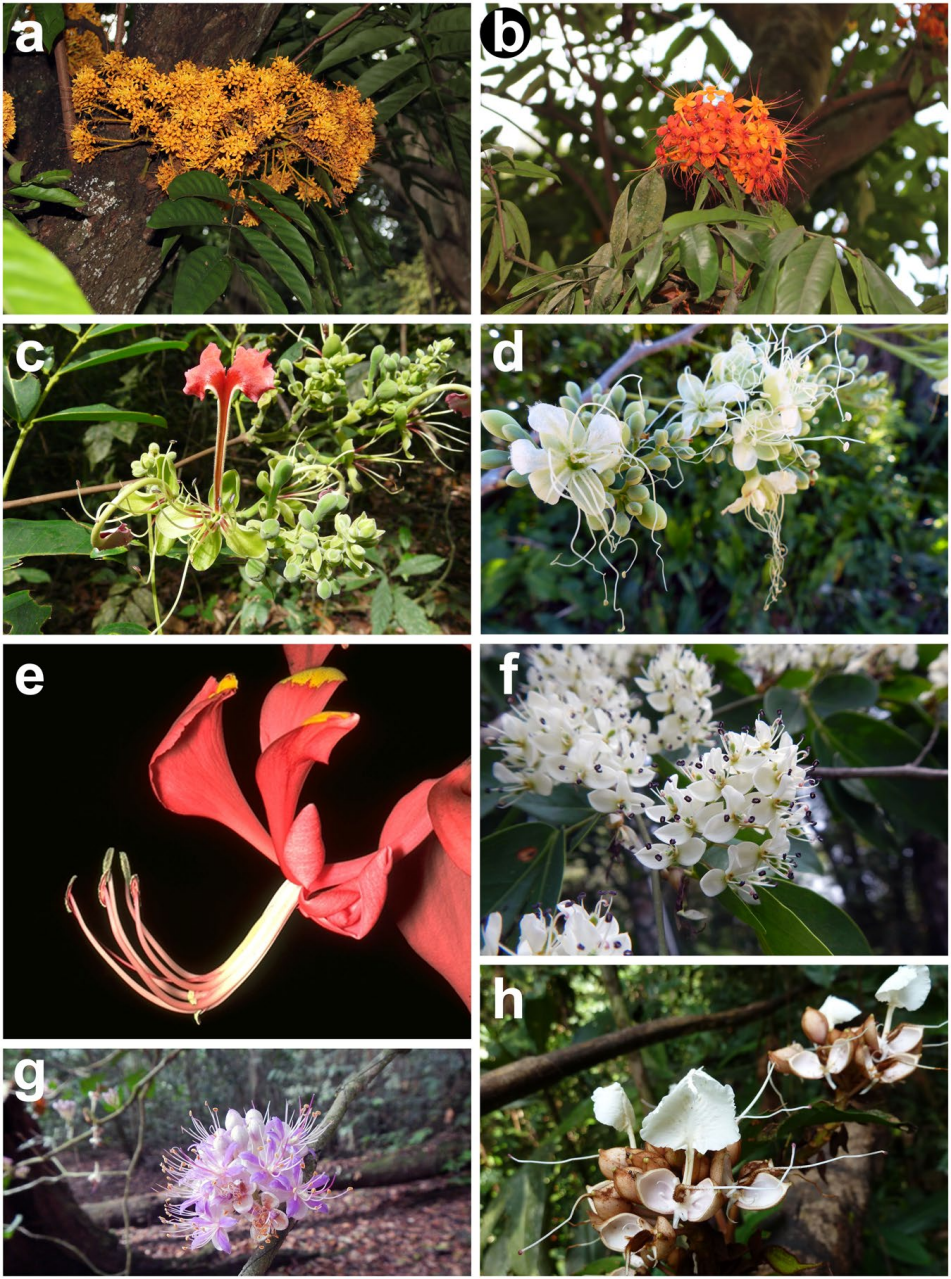
Detarioideae photographs. **(a)** *Saraca cauliflora* (Saraceae). **(b)** *Saraca indica* (Saraceae). **(c)** *Afzelia parviflora* (Afzelieae). **(d)** *Brodriguesia santosii* (Afzelieae). **(e)** *Amherstia nobilis* (Amherstieae). **(f)** *Cryptosepalum tetraphyllum* (Amherstieae). **(g)** *Leonardoxa africana* subsp. *gracilicaulis* (Amherstieae). **(h)** *Gilbertiodendron obliquum* (Amherstieae). — Photos: a & b, M. de la Estrella; c, f-g, X. van der Burgt; d, G.P. Lewis; h, C. Jongkind.

The Saraca clade (Figs 3, 6^30^;) comprises the Asian genera *Endertia*, *Lysidice* and *Saraca*, and is here recognized as a new tribe Saraceae. These genera have in common a tendency to occur in flooded habitats^43^ and together have been consistently resolved as monophyletic in previous phylogenetic studies^29–31^. *Lysidice* and *Endertia* share a characteristic pollen ornamentation consisting of coarse strieae, to short anastomosing striae, to verrucate lirae^87^, and the three genera have bilaterally symmetrical flowers (more radially symmetrical in *Saraca*, which lacks petals) generally with fewer than ten stamens, and staminodes often present (absent in *Endertia*)^4^. *Saraca* is unusual among legumes in having an unique floral homeotic conversion of petal primordia into stamens^88^.

The Afzelia clade (*sensu* Bruneau *et al*.^30^), recognized as the new tribe Afzelieae (Figs 3, 6), is particularly interesting biogeographically and includes three disjunct genera. The monospecific *Brodriguesia* is endemic to the Atlantic forests in Brazil; *Afzelia* is a mainly African genus that is thought to have originated in the savanna but which also includes polyploid species in forest habitats^89^; and *Intsia* is found on both sides of the Indian Ocean and is likely sea-dispersed^2^. *Brodriguesia* has flowers with five almost equally sized petals whereas *Afzelia* and *Intsia* share a similar floral morphology with a large bilobed adaxial petal^2^. Despite these divergent floral patterns, the three genera share leaves with few (and large) leaflets, each with the main vein asymmetrically displaced and a few crateriform glands near the base on the lower surface.

Tribe Amherstieae as here circumscribed was found to be monophyletic by Bruneau *et al*.^30^ with moderate support, and is here strongly supported as monophyletic and sister to Afzelieae (Figs 3 and 4). The strongly supported Brownea clade (Fig. 3), has one poorly supported clade of *Brownea* species occurring as unresolved relative to the other genera and to the remaining Amherstieae clade lineages. The Brownea Group was initially described by Cowan and Polhill^27^ and considered to include 10 neotropical endemic genera. It was subsequently redefined by Bruneau *et al*.^29,31^ to comprise seven genera (*Brownea*, *Browneopsis*, *Macrolobium*, *Paloue*, *Elizabetha*, *Ecuadendron* and *Heterostemon*), with *Brachycylix* and *Paloveopsis* resolved as members of the same clade by Redden *et al*.^39^. However, relationships among the genera of the Brownea clade remain unclear and are currently the focus of further studies (^10^; R. Schley *et al*., unpublished). *Cynometra*, a pantropical genus as currently circumscribed, is well-known to be polyphyletic^90^ and in need of a detailed taxonomic revision (Figs 3 and 4). Some subclades of *Cynometra* are close relatives of the Asian genus *Maniltoa*, while another group of *Cynometra* species are more closely related to *Hymenostegia*, *Talbotiella*, *Loesenera* and *Leonardoxa*. Recently two genera closely related to *Scorodophloeus*^41^, namely *Gabonius* and *Annea*, were described^91,92^ to accommodate three species (two sampled here) that had rendered *Hymenostegia* polyphyletic^41^. As found by Estrella *et al*.^40^, the genus *Gilbertiodendron*, when considered to include *Pellegriniodendron*^72^ is supported as monophyletic, and has been found to form a poorly supported clade with *Librevillea* and *Didelotia* (Fig. 4; Bruneau *et al*.^30^). *Anthonotha*, *Oddoniodendron*, *Isomacrolobium* and *Englerodendron* have been the focus of recent taxonomic treatments^73,74,93,94^ but in our analyses (Fig. 4) their relationships are not clear; and only *Oddoniodendron* is supported as monophyletic. *Berlinia* was monographed by Mackinder & Pennington^15^ who found the genus to be monophyletic in their ITS analysis and sister to a monophyletic *Isoberlinia*^15^. However, generic relationships among *Berlinia* and other Amherstieae clade genera are generally poorly resolved (Figs 4, 6^15,30^). The “babijt” clade was described by Wieringa & Gervais^33^ to group six morphologically close genera, *Brachystegia*, *Aphanocalyx*, *Bikinia*, *Icuria*, *Julbernardia* and *Tetraberlinia* (see also^5,44^), but is not supported as monophyletic in our study (Fig. 4), because it does not include the genus *Microberlinia*, which appears as sister to *Brachystegia* (Fig. 4; Bruneau *et al*.^30^). As suggested by Wieringa & Gervais^33^ this clade likely also contains *Michelsonia* and should then be called “bambijt” clade, but the latter genus could not be properly assessed in this study. The group is characterised by the presence of 10 stamens (nine in *Aphanocalyx libellula*) and in particular bracteoles that have fully taken over the protective function of the reduced to absent sepals and that are partly fused to the hypanthium; the pods have one or two lateral veins.

Although the generic membership of Amherstieae (and the name of the clade) has varied amongst taxonomic treatments^24,27,28,30,95^, there has been general consensus for recognising a cohesive group of genera based on their shared bracteole characteristics. Although the bracteoles in this clade can be morphologically variable, in many genera they are well developed, and are larger than the sepals in bud, and thus perform the protective role normally attributed to the sepals^28^. Certain Amherstieae have spectacularly showy and coloured bracteoles (Fig. 6).

### Gaps in the sampling

Although our study includes a broad sampling of Detarioideae taxa, eight of the 81 genera are missing. Six of these have been sequenced for other loci in previous studies, and can be clearly assigned to the newly designated tribes. *Neoapaloxylon* with three species endemic to Madagascar has been sampled in the broad *matK* LPWG phylogenetic study^3^ and by Fougère-Danezan *et al*.^35,36^ where it was found to be closely related to *Daniellia* and *Brandzeia* in the newly circumscribed Detarieae. *Paloveopsis*, with a single species in Guyana and Brazil, and the monospecific *Brachycylix* endemic to Colombia, were included in the study by Redden *et al*. (^39^; R. Schley *et al*., unpublished), and found to be closely related to *Paloue* and *Ecuadendron*, respectively, both in the Brownea clade of Amherstieae. *Lebruniodendron* with a single species endemic to West Central Africa was resolved as sister to *Crudia* and *Neochevalierodendron*^41^ as is best considered part of Amherstieae, as is *Micklethwaitia*^2,96^, a monospecific genus endemic to Mozambique and previously treated under *Cynometra*, which was found to be closely related to *Gabonius*^2,96^. The monospecific *Michelsonia* from Congo (Kinshasa) was found to belong to the “babijt” clade (*sensu*^33^) within Amherstieae based on a single plastid *psbA-trnH* sequence^44^ confirming the morphological analysis by Wieringa^5^, but the exact relationship of this poorly sampled species remains unresolved. Two genera have never been sequenced because of a lack of material. Nevertheless, *Pseudomacrolobium*, which includes a single species from Congo (Kinshasa), was considered by Mackinder^2^ to be part of Amherstieae, and *Leucostegane* (2 spp. from Malesia), is considered to be closely related to *Saraca* and *Lysidice*^2^ and can confidently be assigned to Saraceae, based on morphological characters.

### Systematic Treatment

Subfamily Detarioideae Burmeist., Handb. Naturgesch.: 319. 1837, emend. LPWG, Taxon 66 (1): 44–77. 2017.

Currently 81 genera and c. 760 species^1–3,43^, almost exclusively tropical with genera present in Central and South America, Africa and South East Asia; and the genus *Schotia* in sub-tropical South Africa.

**Key to Detarioideae Tribes**

1. Leaflets generally with translucent gland dots; cut bark exudes resin………**Detarieae**

1. Leaflets lacking translucent gland dots; cut bark generally not exuding resin………2

2. Bracteoles well-developed (usually persistent), often enveloping the calyx in bud………**Amherstieae**

2. Bracteoles well-developed or not, generally caduceus………3

3. Functional stamens generally fewer than 10, staminodes often present………**Saraceae**

3. Functional stamens generally 10, staminodes absent………4

4. Flower hypanthium shortly tubular, stipe free………**Barnebydendreae**

4. Flower hypanthium shallow, stipe adnate to hypanthium………5

5. Flowers radially symmetrical………**Schotieae**

5. Flowers bilaterally symmetrical………**Afzelieae**

- Tribe **Schotieae** Estrella, L.P. Queiroz & Bruneau, **tribus nov**.

Type: *Schotia* Jacq.

Included genera (1): *Schotia* Jacq. (4 species) (Fig. 5a,b).

Leaflets alternate or opposite, petiolulate, sometimes sessile, lacking translucent gland dots. Flowers radially symmetrical; bracteoles small, caducous, not protecting the bud; sepals 4 (5 initiated but the two adaxial fused at maturity^88^), well developed; petals generally 5, but 1 or more may be reduced or narrow; stamens 10, free or joined at the base; stipe short, adnate to hypanthium. Fruits dehiscent, but the sutural frame persistent. Seeds arillate.

Distribution: tropical and subtropical South Africa, generally in the drier succulent biome^14^.

- Tribe **Barnebydendreae** Estrella, L.P. Queiroz & Bruneau, **tribus nov.**

Type: *Barnebydendron* J.H.Kirkbr.

Included genera (2): *Barnebydendron* J.H. Kirkbr. (1), *Goniorrhachis* Taub. (1) (Fig. 5c,d).

Leaflets opposite, petiolulate, lacking translucent gland dots. Flowers weakly (Goniorrhachis) or strongly (Barnebydendron) bilaterally symmetrical; bracteoles well developed but not showy, caducous to briefly persistent, not protecting the bud; sepals 4, well developed; petals (3-)5, subequal to 2–3 well developed and the remaining petals reduced; stamens 10, free in two whorls (Goniorrhachis) or diadelphus (9 + 1) (Barnebydendron), bent in bud becoming upcurved at anthesis; stipe free in a shortly tubular hypanthium. Fruits indehiscent, samaroid, with a rib on each side parallel to the upper margin. Seeds exarillate.

Distribution: from Central America (Guatemala to Panama) to South America (Bolivia to the Atlantic coast of Brazil). The two species are found in seasonally dry tropical forest, in the succulent biome^14^.

- Tribe **Detarieae** DC., Prodr. 2: 521. 1825. Type: *Detarium* Juss.

Included genera (21): *Augouardia* Pellegr. (1), *Baikiaea* Benth. (4), *Brandzeia* Baill. (1), *Colophospermum* J. Kirk ex J. Léonard (1), *Copaifera* L. (c. 35), *Daniellia* Benn. (10), *Detarium* Juss. (3), *Eperua* Aubl. (14), *Eurypetalum* Harms (2), *Gilletiodendron* Vermoesen (5), *Guibourtia* Benn. (14), *Hardwickia* Roxb. (1), *Hylodendron* Taub. (1), *Hymenaea* L. (14), *Neoapaloxylon* Rauschert (3), *Peltogyne* Vogel (c. 25), *Prioria* Griseb. (including *Gossweilerodendron* Harms, *Kingiodendron* Harms and *Oxystigma* Harms, c. 14 species), *Sindora* Miq. (c. 20), *Sindoropsis* J. Léonard (1), *Stemonocoleus* Harms (1) and *Tessmannia* Harms (c. 12) (Fig. 5e–h).

Leaflets opposite to alternate, petiolulate, often with translucent gland dots, species characterized by the ability to produce bicyclic diterpenes. Flowers with a weak bilateral symmetry; bracteoles small, caducous, not protecting the bud; sepals 4–5 per flower, well developed; petals 0–5, usually equal; stamens generally 10, but sometimes reduced to 3–4 (*Augouardia* and *Stemonocoleus*) or up to 25 (*Colophospermum*), usually several of them partially joined for variable lengths; stipe absent or adnate to hypanthium. Fruits dehiscent or indehiscent. Seeds arillate or exarillate.

Distribution: pantropical, but 11 genera restricted to continental Africa, two restricted to Madagascar, two to Asia and two to the neotropics. Broadly distributed, genera in this tribe tend to occur in wet tropical evergreen forests^14^.

- Tribe **Saraceae** Estrella, L.P. Queiroz & Bruneau, **tribus nov.**

Type: *Saraca* L.

Included genera (4): *Endertia* Steenis & de Wit (1), *Leucostegane* Prain (2), *Lysidice* Hance (2), *Saraca* L. (c. 11) (Fig. 6a,b).

Leaflets opposite or subopposite, petiolulate to sessile, lacking translucent gland dots. Flowers bilaterally symmetrical (radially symmetrical in *Saraca*); bracteoles small to large and showy, usually not protecting the bud; pedicels articulated; sepals 4, well developed, imbricate; petals 0–5, variable in size and shape, generally with 1–3 well developed, remaining vestigial or absent; stamens 2 [3–8(−10) in *Saraca*], free, usually 3–8 staminodes also present; ovary stipe free to adnate to the hypanthium wall. Fruits dehiscent with twisting valves. Seeds exarillate.

Distribution: from Indo-China to Malesia, extending to the Pacific islands, generally in lowland tropical forest, within the rainforest biome^14^.

- Tribe **Afzelieae** Estrella, L.P. Queiroz & Bruneau, **tribus nov.**

Type: *Afzelia* Sm.

Included genera (3): *Afzelia* Sm. (c. 11), *Brodriguesia* R.S. Cowan (1), *Intsia* Thouars (3) (Fig. 6c,d).

Leaflets opposite, petiolulate, lacking translucent gland dots. Flowers bilaterally symmetrical; bracteoles well developed, caducous, not protecting the flower; sepals 4, well developed, imbricate, only 2 visible in bud; petals 5, one large petal and 4 reduced (Afzelia and Intsia) or 5 well developed (Brodriguesia); stamens 3 (Intsia), 7(−9) (Afzelia) or 10 (Brodriguesia), free or basally connate; stipe adnate to hypanthium. Fruits dehiscent but valves not becoming twisted. Seeds with a cupular or annular aril, or aril-like structure.

Distribution: pantropical. *Intsia* and *Brodriguesia* are distributed within the rainforest biome, meanwhile *Afzelia* species appear within rainforest and the grassland biomes^14,89^.

- Tribe **Amherstieae** Benth., J. Bot. (Hooker) 2: 73. 1840. Type: Amherstia Wall.

Included genera (50): *Amherstia* Wall. (1), *Annea* Mackinder & Wieringa (2), *Anthonotha* P. Beauv. (c. 30), *Aphanocalyx* Oliver (14), *Berlinia* Sol. ex Hook. f. (c. 17), *Bikinia* Wieringa (10), *Brachycylix* (Harms) R.S. Cowan (1), *Brachystegia* Benth. (c. 26), *Brownea* Jacq. (c. 12), *Browneopsis* Huber (6), *Crudia* Schreb. (c. 55), *Cryptosepalum* Benth. (c. 11), *Cynometra* L. (c. 90), *Dicymbe* Spruce ex Benth. & Hook. f. (c. 20), *Didelotia* Baill. (c. 12), *Ecuadendron* D.A. Neill (1), *Elizabetha* Schomb. ex Benth. (c. 11), *Englerodendron* Harms (1), *Gabonius* Wieringa & Mackinder (1), *Gilbertiodendron* J. Léonard (c. 30), *Heterostemon* Desf. (7), *Humboldtia* Vahl (6), *Hymenostegia* (Benth.) Harms (c. 16), *Icuria* Wieringa (1), *Isoberlinia* Craib & Stapf ex Holland (c. 5), *Isomacrolobium* Aubrév. & & Pellegr. (12), *Julbernardia* Pellegr. (c. 11), *Lebruniodendron* J. Léonard (1), *Leonardoxa* Aubrév. (1), *Librevillea* Hoyle (1), *Loesenera* Harms (4), *Macrolobium* Schreb. (c. 80), *Maniltoa* Scheff. (c. 25), *Michelsonia* Hauman (1), *Micklethwaitia* G.P. Lewis & Schrire (1), *Microberlinia* A. Chev. (2), *Neochevalierodendron* J. Léonard (1), *Normandiodendron* J. Léonard (2), *Oddoniodendron* De Wild. (c. 3), *Paloue* Aubl. (4), *Paloveopsis* R.S. Cowan (1), *Paramacrolobium* J.Léonard (1), *Plagiosiphon* Harms (5), *Polystemonanthus* Harms (1), *Pseudomacrolobium* Hauman (1), *Scorodophloeus* Harms (3), *Talbotiella* Baker f. (8), *Tamarindus* L. (1), *Tetraberlinia* (Harms) Hauman (7) and *Zenkerella* Taub. (c. 5) (Fig. 6e–h).

Leaflets opposite or alternate, petiolulate to sessile, lacking translucent gland dots. Flowers bilaterally to radially symmetrical; bracteoles variable, but often well developed, and becoming larger than the sepals/calyx in flower bud; sepals (0-) 4–5 (−10), occasionally in some genera the two adaxial ones (partly) joined; petals variable, (0-) 5 (−6) often one or two petals enlarged, the remaining ones reduced or absent; stamens extremely variable, generally 3–10 but up to 80 (e.g., in *Maniltoa*), free or basally connate, often diadelphus, sometimes staminodia also present; stipe of the ovary free or adnate to hypanthium wall. Fruits mostly explosively dehiscent, or indehiscent (*Tamarindus*). Seeds exarillate.

Distribution: predominantly pantropical, but with 34 genera restricted to continental Africa and nine to Central and South America. genera in this tribe tend to occur in wet tropical evergreen forests^14^.

### Data availability

The sequences used in this study are available for download from the GenBank database of the National Center for Biotechnology Information (http://www.ncbi.nlm.nih.gov/genbank/). See Supplementary Appendix I for the accession numbers of all samples included.

## Electronic supplementary material

Supplementary Information
