# Supplementary material for: A new phylogeny-based tribal classification of subfamily Detarioideae, an early branching clade of florally diverse tropical arborescent legumes
Source: Sci Rep. 2018 May 2;8:6884. doi: 10.1038/s41598-018-24687-3 (PMC5932001; doi:10.1038/s41598-018-24687-3)

A new phylogeny-based tribal classification of subfamily Detarioideae, an early branching clade of florally diverse tropical arborescent legumes.

Manuel de la Estrella, Félix Forest, Bente Klitgård, Gwilym P. Lewis, Barbara A. Mackinder, Luciano P. de Queiroz, Jan J. Wieringa & Anne Bruneau

Appendix I. Voucher information of accessions used in this study (–– denotes absence of marker).

SPECIES	Specimen (Herbarium)	matK-trnK	rpL16	trnG-trnG2G	ITS
Adenolobus garipensis	Leistuer 246 (K)	EU361844	KY313125	KY312861	KY306484
Afzelia africana	Jongkind 2440 (WAG)	KX161926	KY313126	KY312862	KY306485
Afzelia bella	Breteler 13120 (WAG)	KX161927	MG949387	MG949575	––
Afzelia bella	Breteler 14446 (WAG)	KX161928	KY313127	KY312863	KY306486
Afzelia bipindensis	Wieringa 5828 (WAG)	KX161929	KY313128	KY312864	KY306487
Afzelia bipindensis	Breteler 15405 (WAG)	EU361847	MG949388	MG949576	MG949314
Afzelia quanzensis	Herendeen 9-XII-97-4 (US)	KX161930	KY313129	––	KY306488
Amherstia nobilis	Baker 490 (K)	EU361849	KY313132	KY312865	KY306489
Amherstia nobilis	Breteler 13507 (WAG)	KX161931	KY313133	KY312866	KY306490
Amherstia nobilis	Bruneau sn (K)	KX161932	MG949389	MG949577	MG949321
Annea afzelii	Andel 4244 (WAG)	KX161933	KY313134	KY312867	KY306491
Anthonotha brieyi	Wieringa 6082 (WAG)	KX161935	KY313135	KY312868	KY306492
Anthonotha crassiflora	Wieringa 6391 (WAG)	KX161936	KY313136	KY312869	KY306493
Anthonotha fragrans	Breteler 15379 (WAG)	KX161937	KY313137	––	KY306494
Anthonotha macrophylla	Wieringa 2996 (WAG)	EU361853	MG949390	MG949579	AF513653
Anthonotha macrophylla	Wieringa 5463 (WAG)	KX161938	MG949391	MG949580	MG949374
Anthonotha sp	Bruneau 1059 (MT)	KX161934	MG949392	MG949578	MG949317
Anthonotha sp	Sosef 1489 (WAG)	KX161939	MG949393	MG949581	MG949353
Anthonotha stipulacea	Walters 591 (WAG)	KX161940	KY313140	KY312871	KY306496
Anthonotha trunciflora	Breteler 14355 (WAG)	EU361852	KY313139	KY312870	KY306495
Anthonotha xanderi	Wieringa 5861 (WAG)	KX161941	KY313141	KY312872	KY306497
Aphanocalyx cynometroides	Wieringa 2355 (WAG)	EU361855	KY313142	KY312873	KY306498
Aphanocalyx djumaensis	Breteler 13056 (WAG)	EU361856	KY313143	KY312874	AF513655
Aphanocalyx ledermannii	Wieringa 2263 (WAG)	KX161943	MG949394	MG949586	AF513659
Aphanocalyx ledermannii	Wieringa 1310 (WAG)	KX161942	KY313144	KY312875	AF513660
Aphanocalyx margininervatus	Breteler 12346 (WAG)	––	KY313145	KY312876	AF513661
Aphanocalyx microphyllus compactus	Breteler 13356 (WAG)	KX161948	KY313146	KY312878	AF513662
Aphanocalyx microphyllus microphyllus	Ewango 852 (WAG)	KX161944	KY313147	KY312877	AF513664
Aphanocalyx microphyllus microphyllus	Valkenburg 3067 (WAG)	KX161945	MG949395	MG949587	MG949355
Aphanocalyx microphyllus microphyllus	Wieringa 2559 (WAG)	KX161946	MG949396	MG949588	MG949360
Aphanocalyx microphyllus microphyllus	Wieringa 3103 (WAG)	KX161947	MG949397	MG949589	MG949364
Aphanocalyx obscurus	Wieringa 1541 (WAG)	KX161949	KY313148	KY312879	AF513666
Aphanocalyx pectinatus	Wieringa 3102 (WAG)	KX161950	KY313149	––	AF513668
Augouardia letestui	Breteler 14667 (WAG)	EU361862	KY313130	KY312880	AY955796
Augouardia letestui	Wieringa 2898 (WAG)	KX161951	KY313131	KY312881	KY306499
Augouardia letestui	Wieringa 2974 (WAG)	KX161952	––	––	MG949363
Baikiaea insignis	Breteler 12655 (WAG)	KX161953	MG949398	MG949590	––
Baikiaea insignis	Bruneau sn (K)	EU361863	KY313151	KY312883	AY955811
Baikiaea insignis	Breteler 14464 (WAG)	KX161954	KY313150	KY312882	KY306500
Baikiaea plurijuga	Rodin 4507 (US)	––	KY313152	KY312884	KY306501
Barnebydendron riedelii	Hughes 415 (K)	KX161956	MG949399	MG949591	––
Barnebydendron riedelii	Brammall sn (K)	EU361868	––	––	AY955777
Barnebydendron riedelii	Hughes 1104 (K)	KX161955	KY313153	KY312885	KY306502
Barnebydendron riedelii	Lewis 1224 (K)	KX161957	KY313154	––	KY306503
Barnebydendron riedelii	Lewis 1615 (K)	––	MG949400	––	MG949340
Berlinia bracteosa	Wieringa 6414 (WAG)	KX161960	KY313155	KY312886	KY306504
Berlinia bracteosa	Breteler 14719 (WAG)	KX161958	MG949401	MG949592	MG949310
Berlinia bracteosa	Rickson sn (OSC)	KX161959	MG949402	MG949593	MG949349
Berlinia brunnelii	Sosef 2248 (WAG)	KX161961	KY313156	KY312887	KY306505
Berlinia confusa	Breteler 15455 (WAG)	EU361879	KY313157	KY312888	KY306506
Berlinia congolensis	Valkenburg 3106 (WAG)	KX161962	KY313158	KY312889	KY306507

Berlinia congolensis	Breteler 15425 (WAG)	EU361881	MG949403	MG949594	MG949315
Berlinia grandiflora	Jongkind 2516 (WAG)	EU361882	KY313159	KY312890	KY306508
Berlinia viridicans	Breteler 14485 (WAG)	KX161964	MG949404	MG949595	MG949306
Bikinia breynii	Breteler 14198 (WAG)	KX161965	KY313160	KY312891	KY306509
Bikinia cf. *pellegrinii*	Wieringa 2828 (WAG)	KX161974	MG949406	MG949585	AF513685
Bikinia coriacea	Breteler 12972 (WAG)	KX161966	KY313161	KY312892	AF513675
Bikinia grisea	Wieringa 6210 (WAG)	KX161967	KY313162	KY312893	KY306510
Bikinia letestui	Wieringa 5554 (WAG)	KX161968	KY313163	KY312894	KY306511
Bikinia letestui	Wilde sn (WAG)	KX161969	MG949405	MG949582	MG949384
Bikinia media	Wieringa 6211 (WAG)	KX161971	KY313164	KY312895	––
Bikinia media	Wieringa 3125 (WAG)	KX161970	MG949407	MG949583	––
Bikinia pellegrini	Wieringa 6138 (WAG)	KX161972	KY313165	KY312896	KY306512
Bikinia pellegrinii	Breteler 13305 (WAG)	KX161973	MG949408	MG949584	––
Bikinia sp	Wieringa 4711 (WAG)	KX161975	MG949409	––	MG949368
Brachystegia boehmii	Gerreau 3054 (NY)	EU361886	KY313166	––	KY306513
Brachystegia bussei	Herendeen 20-XII-97-3 (US)	KX161976	KY313167	KY312897	KY306514
Brachystegia floribunda	Herendeen 21-XII-97-1 (US)	KX161977	KY313168	KY312898	KY306515
Brachystegia laurentii	Wieringa 4529 (WAG)	KX161978	KY313169	KY312899	KY306516
Brachystegia leonensis	Breteler 13353 (WAG)	KX161979	KY313170	KY312900	KY306517
Brachystegia longifolia	Herendeen 21-XII-97-2 (US)	KX161980	KY313171	KY312901	AF513687
Brachystegia mildbraedii	JJJFE de Wilde 11718 (WAG)	KX161981	KY313172	KY312902	AF513688
Brachystegia spiciformis	Herendeen 9-XII97-9 (US)	EU361888	KY313173	KY312903	KY306518
Brandzeia filicifolia	Morat 949 (P)	KX161982	MG949410	MG949596	AY955794
Brandzeia filicifolia	Du Puy 403 (K)	EU361870	KY313174	KY312904	KY306519
Brodriguesia santosii	Jardim 2652 (MO)	KX161983	KY313175	KY312905	––
Brownea ariza	Fougere sn (MT)	KX161984	KY313177	KY312906	KY306520
Brownea ariza	Wieringa 4167 (WAG)	KX161985	MG949411	MF987562	MF987598
Brownea capitella	Fougere sn (MT)	KX161986	KY313178	KY312907	KY306521
Brownea coccinea	Fougere 33 (MT)	KX161988	KY313179	KY312908	KY306522
Brownea coccinea	Clarke 4139 (US)	EU361891	MG949413	––	MF987600
Brownea coccinea	Baker 600 (K)	KX161987	MG949412	––	MF987599
Brownea grandiceps	Fougere sn (MT)	KX161990	KY313180	KY312909	KY306523
Brownea grandiceps	Klitgaard 67040 (K)	KX161989	MG949414	––	MF987601
Brownea latifolia	Steyermark 88845 (NY)	KX161991	KY313181	––	KY306524
Brownea leucantha	Klitgaard 666 (K)	KX161992	KY313176	KY312910	KY306525
Brownea sp	Fougere 11 (MT)	KX161993	MG949415	MF987561	MF987602
Brownea sp	Pitman 5721 (K)	KX161994	MG949416	MF987570	MF987603
Browneopsis disepala	Klitgaard 67032 (K)	KX161995	KY313182	KY312911	KY306526
Browneopsis ucayalina	Neill 6376 (K)	KX161996	KY313183	––	KY306527
Cercis canadensis	Stevees sn (MT)	KX161997	KY313184	KY312912	KY306528
Colophospermum mopane	Bingham 11341 (K)	EU361915	KY313185	KY312913	AY955788
Copaifera mildbraedii	Wieringa 2356 (WAG)	––	MG949563	MG949734	––
Copaifera mildbraedii	Breteler 15025 (WAG)	EU361917	KY313186	KY312914	AY955814
Copaifera mildbraedii	Breteler 13149 (WAG)	KX161998	MG949417	MG949597	MG949299
Copaifera officinalis	Fougere 27 (MT)	EU361918	KY313187	KY312915	AY955816
Copaifera religiosa	Wieringa 5501 (WAG)	KX161999	KY313188	KY312916	––
Copaifera salikounda	Breteler 13383 (WAG)	EU361919	KY313189	KY312917	AY955815
Crudia aequalis	Redden 3498 (US)	KX162000	KY313190	KY312918	KY306529
Crudia aromatica	Redden 1105 (US)	KX162001	KY313191	KY312919	KY306530
Crudia bracteata	Forest 131 (K)	KX162002	KY313192	KY312920	KY306531
Crudia caudata	Herendeen 2-V-99-3 (US)	KX162003	KY313193	KY312921	KY306532
Crudia choussyana	Hughes 1249 (K)	EU361921	KY313194	––	KY306533
Crudia gabonensis	Breteler 13770 (WAG)	KX162004	––	KY312922	KY306534
Crudia klainei	Breteler 14603 (WAG)	KX162005	MG949418	MG949598	––
Crudia klainei	Wieringa 2104 (WAG)	KX162006	KY313195	––	KY306535
Crudia lanceolata	Herendeen 27-IV-99-1 (US)	KX162007	KY313196	KY312923	KY306536
Crudia lanceolata	Herendeen 27-IV-99-5 (US)	KX162008	MG949419	MG949599	MG949331
Crudia oblonga	Klitgaard 3710 (K)	KX162009	KY313197	KY312924	KY306537
Crudia sp	Gray 8932 (QRS)	KX162010	MG949420	MG949600	MG949324
Cryptosepalum pellegrinianum	Breteler 15206 (WAG)	KX162011	KY313198	KY312925	KY306538

Cryptosepalum pellegrinianum	Breteler 15241 (WAG)	KX162012	MG949421	MG949601	MG949312
Cryptosepalum pellegrinianum	Wieringa 6115 (WAG)	KX162013	MG949422	MG949602	MG949377
Cryptosepalum staudtii	Leal 49 (WAG)	EU361923	KY313199	––	KY306539
Cryptosepalum tetraphyllum	Breteler 13375 (WAG)	KX162014	KY313200	KY312926	KY306540
Cynometra crassifoza	Klitgaard 325 (K)	KX162016	KY313201	KY312927	KY306541
Cynometra hankei	Wieringa 5930 (WAG)	KX162017	KY313202	KY312928	KY306542
Cynometra iripa	Herendeen 4-V-99-1 (US)	KX162019	KY313203	KY312929	KY306543
Cynometra iripa	Gray 8933 (QRS)	KX162018	MG949423	MG949603	MG949325
Cynometra letestui	Breteler 13966 (WAG)	KX162020	––	––	MG949304
Cynometra lujae	Valkenburg 3006 (WAG)	KX162021	KY313204	KY312930	KY306544
Cynometra malaccensis	Herendeen 29-IV-99-7 (US)	KX162023	KY313205	KY312931	KY306545
Cynometra malaccensis	Herendeen 27-IV-99-4 (US)	KX162022	MG949424	MG949604	MG949330
Cynometra mannii	Breteler 14421 (WAG)	KX162024	KY313206	KY312932	KY306546
Cynometra mannii	Breteler 15534 (WAG)	KX162025	MG949425	MG949605	MG949316
Cynometra ramiflora	Herendeen 24-IV-1995 (US)	KX162028	KY313208	KY312934	KY306548
Cynometra ramiflora	Breteler 13510 (WAG)	KX162027	MG949426	MG949606	MG949301
Cynometra schlechteri	Breteler 13140 (WAG)	KX162029	KY313209	KY312935	KY306549
Cynometra sp	Herendeen 16-XII-97-1 (US)	EU361924	MG949429	MG949609	MG949332
Cynometra sp	Bruneau 1361 (K)	KX162030	MG949427	MG949607	MG949319
Cynometra sp	Herendeen 17-XII-97-4 (US)	KX162031	MG949430	MG949610	MG949333
Cynometra sp	Herendeen 17-XII-97-7 (US)	KX162032	MG949431	MG949611	MG949334
Cynometra sp	Bruneau 1364 (K)	––	MG949428	MG949608	MG949320
Daniellia klainei	Breteler 15223 (WAG)	EU361927	KY313210	KY312936	AY955791
Daniellia klainei	Sosef 2451 (WAG)	KX162033	MG949432	––	MG949354
Daniellia klainei	Valkenburg 3151 (WAG)	KX162034	MG949433	MG949612	MG949356
Daniellia oblonga	Breteler 11046 (WAG)	––	––	MG949613	MG949293
Daniellia ogea	Bruneau sn (K)	KX162035	KY313211	KY312937	AY955790
Daniellia ogea	Bruneau sn (K)	KX162036	KY313212	KY312938	KY306550
Daniellia ogea	Adjakidjè 3985 (WAG)	––	MG949434	MG949614	MG949290
Daniellia oliveri	Jongkind 2101b (WAG)	KX162038	MG949435	––	AY955792
Daniellia oliveri	Jongkind 1953 (WAG)	KX162037	KY313213	KY312939	KY306551
Daniellia oliveri	Kuppers 1576	KX162039	MG949436	MG949615	MG949337
Daniellia pilosa	Wieringa 1462 (WAG)	KX162042	KY313214	KY312940	KY306552
Daniellia pilosa	Halle 4610 (WAG)	KX162040	MG949437	MG949616	MG949327
Daniellia pilosa	Hombert 502 (WAG)	KX162041	MG949438	MG949617	MG949335
Daniellia pynaertii	Harris 8528 (WAG)	KX162043	KY313215	KY312941	KY306553
Daniellia soyauxii	Wieringa 2614 (WAG)	––	MG949439	MG949618	AY955793
Daniellia soyauxii	Wieringa 6064 (WAG)	KX162044	KY313216	KY312942	KY306554
Daniellia thurifera	Clark 183 (K)	KX162045	––	KY312943	KY306555
Detarium macrocarpum	Breteler 14752 (WAG)	KX162046	KY313217	––	AY955817
Dicymbe corymbosa	Redden 1345 (US)	KX162047	KY313218	––	KY306556
Dicymbe jenanni	Redden 2473 (US)	KX162048	KY313219	––	KY306557
Dicymbe levi	Redden 1367 (US)	KX162050	KY313220	KY312944	KY306558
Dicymbe levi	Redden 1253 (US)	KX162049	MG949440	MG949619	MG949344
Dicymbe sp	Redden 1205 (US)	KX162051	MG949441	MG949620	MG949342
Dicymbe sp	Redden 1215 (US)	KX162052	MG949442	MG949621	MG949343
Dicymbe sp	Redden 1368 (US)	KX162053	MG949443	MG949622	MG949345
Dicymbe sp	Redden 1388 (US)	KX162054	MG949444	MG949623	MG949346
Dicymbe sp	Redden 2282 (US)	KX162055	MG949445	MG949624	MG949347
Dicymbe sp	Redden 3214 (US)	KX162056	MG949446	MG949625	MG949348
Didelotia africana	Sosef 2509 (WAG)	KX162058	KY313221	KY312945	KJ777183
Didelotia africana	Wieringa 3549 (WAG)	KX162059	MG949448	MG949627	KJ777184
Didelotia africana	Breteler 14596 (WAG)	KX162057	MG949447	MG949626	MG949308
Didelotia letouzeyii	Wieringa 5065 (WAG)	KX162060	MG949449	MG949628	KJ777185
Didelotia letouzeyii	Wieringa 6120 (WAG)	KX162061	MG949450	MG949629	KJ777186
Didelotia letouzeyii	Wieringa 6187 (WAG)	KX162062	KY313222	KY312946	KJ777187
Didelotia minutiflora	Wieringa 4819 (WAG)	KX162063	KY313223	KY312947	KJ777188
Didelotia minutiflora	Wieringa 2424 (WAG)	––	MG949451	MG949630	MG949359
Didelotia unifoliata	Sosef 2517 (WAG)	KX162064	KY313224	KY312948	KJ777189
Duparquetia orchidacea	Barbera 104 (MA)	KX162065	KY313225	KY312949	KY306559

Duparquetia orchidacea	Wieringa 4351 (WAG)	KX162066	KY313226	KY312950	KY306560
Ecuadendron acostasolisianum	Lewis 2876 (K)	KX162067	KY313227	KY312951	KY306561
Ecuadendron acostasolisianum	Neill 10437 (MO)	EU361938	KY313228	KY312952	KY306562
Ecuadendron acostasolisianum	Clarke 6220 (US)	––	MG949452	MF987571	MF987604
Elizabetha coccinea	Redden 1181 (US)	KX162068	KY313229	––	KY306563
Elizabetha fanshawei	Redden 1733 (US)	KX162069	KY313230	––	KY306564
Elizabetha grahamiae	Redden 3090 (US)	KX162070	KY313231	KY312953	KY306565
Elizabetha grahamiae	Redden 3307 (US)	KX162071	KY313232	KY312954	KY306566
Elizabetha macrostachya	Redden 3714 (US)	KX162072	KY313233	KY312955	FJ817516
Elizabetha paraensis	Breteler 13791 (WAG)	EU361941	KY313234	KY312956	KY306567
Elizabetha princeps	Redden 3692 (US)	KX162073	KY313235	KY312957	FJ817519
Endertia spectabilis	Sosef 364 (WAG)	EU361943	KY313236	KY312958	KY306568
Englerodendron conchyliophorum	Sosef 1151 (WAG)	KX162074	KY313237	KY312959	KY306569
Englerodendron gabunense	Breteler 11174 (WAG)	KX162075	KY313138	KY312960	KY306570
Englerodendron gabunense	Wieringa 6213 (WAG)	KX162076	MG949453	MG949632	MG949379
Englerodendron korupense	Burgt 741 (WAG)	KX162077	KY313238	KY312961	KY306571
Englerodendron usambarense	Herendeen 17-XII-97-2 (US)	EU361944	KY313239	KY312962	KY306572
Eperua falcata	Cable 1189 (K)	KX162078	KY313240	KY312963	AY955805
Eperua grandiflora	Clarke 7639 (US)	EU361946	KY313241	KY312964	AY955807
Eperua sp	Bruneau 1154 (K)	KX162079	MG949532	MG949633	MG949318
Erythrophleum suaveolens	Herendeen 11-XII-97-2 (US)	KX162080	KY313242	KY312965	KY306573
Eurypetalum tessmannii	Breteler 14165 (WAG)	EU361950	KY313243	KY312966	AY955804
Eurypetalum tessmannii	Breteler 12197 (WAG)	KX162081	MG949454	MG949634	MG949295
Eurypetalum unijugum	Letouzey 9787 (P)	––	––	MG949635	AY955803
Eurypetalum unijugum	Wieringa 2122 (WAG)	KX162082	KY313244	KY312967	KY306574
Gabonius ngouniensis	Wieringa 2579 (WAG)	EU361977	KY313302	KY312968	KY306575
Gabonius ngouniensis	Wieringa 5094 (WAG)	KX162084	KY313245	––	KY306576
Gabonius ngouniensis	Breteler 14351 (WAG)	KX162083	MG949522	MG949636	MG949305
Gilbertiodendron aylmeri	Burgt 1458 (WAG)	KX162086	MG949455	MG949638	KJ777190
Gilbertiodendron aylmeri	Jongkind 7003 (WAG)	KX162087	KY313248	KY312969	KJ777191
Gilbertiodendron bil	Jongkind 8775 (WAG)	KX162088	KY313249	KY312970	KJ777192
Gilbertiodendron bilineatum	Jongkind 5437 (WAG)	KX162089	KY313250	KY312971	KJ777193
Gilbertiodendron brachystegioides	Breteler 14983 (WAG)	KX162091	MG949457	MG949639	KJ777194
Gilbertiodendron brachystegioides	Burgt 801 (WAG)	KX162092	MG949458	MG949640	KJ777195
Gilbertiodendron brachystegioides	Wieringa 2242 (WAG)	KX162094	MG949460	MG949642	KJ777196
Gilbertiodendron brachystegioides	Wieringa 6226 (WAG)	KX162155	MG949498	MG949682	KJ777197
Gilbertiodendron breteleri	Wieringa 6099 (WAG)	KX162095	MG949461	MG949643	KJ777253
Gilbertiodendron breteleri	Breteler 14250 (WAG)	KX162090	KY313269	KY312972	KJ777258
Gilbertiodendron breteleri	Wieringa 6190 (WAG)	KX162096	MG949462	––	KJ777259
Gilbertiodendron breteleri	Wieringa 7559 (WAG)	––	MG949499	MG949683	MG949380
Gilbertiodendron demonstrans	Burgt 731 (WAG)	KX162097	MG949465	MG949644	KJ777202
Gilbertiodendron demonstrans	Wieringa 2202 (WAG)	KX162098	KY313252	KY312973	KJ777203
Gilbertiodendron demonstrans	Wieringa 2362 (WAG)	KX162099	––	MG949645	KJ777204
Gilbertiodendron dewevrei	Andel 4045 (WAG)	KX162100	KY313246	KY312974	KJ777205
Gilbertiodendron dewevrei	Burgt 783 (WAG)	KX162101	MG949466	MG949646	KJ777206
Gilbertiodendron dewevrei	Wieringa 3552 (WAG)	KX162102	MG949467	MG949647	KJ777208
Gilbertiodendron dewevrei	Wieringa 6091 (WAG)	KX162103	MG949468	MG949648	KJ777209
Gilbertiodendron dewevrei	Wieringa 6261 (WAG)	KX162104	MG949469	MG949649	KJ777210
Gilbertiodendron dewevrei	Wieringa 7555 (WAG)	KX162105	KY313253	KY312975	KY306577
Gilbertiodendron diphyllum	Andel 3502 (WAG)	KX162106	KY313254	KY312976	KJ777211
Gilbertiodendron diphyllum	Andel 4111 (WAG)	KX162107	MG949503	MG949650	KJ777212
Gilbertiodendron diphyllum	Breteler 13402 (WAG)	KX162108	––	MG949651	KJ777213
Gilbertiodendron diphyllum	Breteler 13734 (WAG)	KX162109	MG949470	MG949652	KJ777214
Gilbertiodendron diphyllum	Jongkind 9311 (WAG)	KX162110	MG949471	MG949653	KJ777215
Gilbertiodendron diphyllum	Louis 2481 (WAG)	KX162111	KY313255	KY312977	KJ777216
Gilbertiodendron diphyllum	Parren 306 (WAG)	––	MG949472	––	KJ777217
Gilbertiodendron diphyllum	Breteler 10782 (WAG)	KX162114	MG949473	MG949656	KJ777218
Gilbertiodendron diphyllum	Wieringa 4105 (WAG)	KX162112	MG949474	MG949654	KJ777219
Gilbertiodendron diphyllum	Wieringa 6218 (WAG)	KX162113	MG949504	MG949655	KJ777220
Gilbertiodendron ecoukense	Bongou 144 (WAG)	KX162115	MG949475	MG949657	KJ777199

Gilbertiodendron ecoukense	Bongou 387 (WAG)	KX162116	KY313256	KY312978	KJ777201
Gilbertiodendron ecoukense	Wieringa 7723 (WAG)	KX162170	MG949508	MG949692	MG949381
Gilbertiodendron grandistipulatum	Breteler 10428 (WAG)	KX162117	KY313257	KY312979	KJ777221
Gilbertiodendron grandistipulatum	White 1118 (WAG)	KX162118	––	––	KJ777222
Gilbertiodendron grandistipulatum	Wieringa 4687 (WAG)	KX162119	MG949476	MG949658	KJ777223
Gilbertiodendron imeoense	Breteler 9974 (WAG)	KX162120	KY313258	––	KJ777224
Gilbertiodendron ivorense	Jongkind 9210 (WAG)	KX162121	KY313251	KY312980	KJ777225
Gilbertiodendron ivorense	Stoop 19 (WAG)	KX162122	––	MG949659	KJ777226
Gilbertiodendron jongkindii	Jongkind 4502 (WAG)	KX162123	KY313259	KY312981	KJ777227
Gilbertiodendron jongkindii	Jongkind 9350 (WAG)	KX162124	MG949463	MG949660	KJ777228
Gilbertiodendron klainei	Wieringa 1239 (WAG)	KX162125	MG949477	––	KJ777229
Gilbertiodendron klainei	Wieringa 2443 (WAG)	KX162126	KY313260	KY312982	KJ777230
Gilbertiodendron limba	Jongkind 1438 (WAG)	KX162127	KY313261	KY312983	KJ777231
Gilbertiodendron maximum	Wieringa 1315 (WAG)	KX162152	MG949495	MG949679	KJ777262
Gilbertiodendron mayombense	Burgt 595 (WAG)	KX162129	MG949464	MG949661	KJ777198
Gilbertiodendron mayombense	Breteler 14147 (WAG)	KX162128	KY313262	KY312984	KJ777232
Gilbertiodendron mayombense	Wieringa 1475 (WAG)	KX162130	MG949478	MG949662	KJ777233
Gilbertiodendron mayombense	Wieringa 6191 (WAG)	KX162131	MG949479	MG949663	KJ777260
Gilbertiodendron newberyii	Burgt 774 (WAG)	KX162132	KY313263	KY312985	KJ777234
Gilbertiodendron newberyii	Burgt 776 (WAG)	KX162133	MG949480	MG949664	KJ777235
Gilbertiodendron obliquum	Jongkind 9972 (WAG)	KX162134	KY313264	KY312986	KJ777236
Gilbertiodendron ogoouense	Breteler 10258 (WAG)	KX162135	MG949481	MG949665	KJ777237
Gilbertiodendron ogoouense	Breteler 11450 (WAG)	KX162136	MG949482	MG949666	KJ777238
Gilbertiodendron ogoouense	Breteler 11524 (WAG)	––	MG949483	MG949667	KJ777239
Gilbertiodendron ogoouense	Burgt 775 (WAG)	KX162137	MG949484	MG949668	KJ777240
Gilbertiodendron ogoouense	Mpandzou 44 (WAG)	KX162150	MG949485	MG949677	KJ777241
Gilbertiodendron ogoouense	Schoenmaker 70 (WAG)	––	MG949486	MG949669	KJ777242
Gilbertiodendron ogoouense	Wieringa 1316 (WAG)	KX162139	MG949487	MG949670	KJ777243
Gilbertiodendron ogoouense	Wieringa 2777 (WAG)	KX162140	MG949488	MG949671	KJ777244
Gilbertiodendron preussii	Andel 4118 (WAG)	KX162142	KY313266	KY312989	KJ777245
Gilbertiodendron preussii	Burgt 794 (WAG)	KX162143	KY313267	KY312990	KJ777246
Gilbertiodendron preussii	Wieringa 1600 (WAG)	KX162144	MG949489	MG949672	KJ777247
Gilbertiodendron quinquejugum	Bongou 105 (WAG)	KX162148	MG949492	MG949675	KJ777254
Gilbertiodendron quinquejugum	Bongou 415 (WAG)	KX162141	KY313247	KY312988	KJ777255
Gilbertiodendron quinquejugum	Valkenburg 2635 (WAG)	KX162138	KY313265	KY312987	KJ777256
Gilbertiodendron quinquejugum	Valkenburg 2636 (WAG)	KX162093	MG949459	MG949641	KJ777257
Gilbertiodendron robynsianum	Jongkind 4525 (WAG)	KX162145	KY313268	KY312991	KJ777248
Gilbertiodendron robynsianum	Jongkind 4942 (WAG)	KX162146	MG949490	MG949673	KJ777249
Gilbertiodendron robynsianum	Jongkind 5644 (WAG)	KX162147	MG949491	MG949674	KJ777250
Gilbertiodendron scutatum	Wieringa 5057 (WAG)	KX162153	MG949456	MG949680	KJ777252
Gilbertiodendron sp	Jongkind 8884 (WAG)	KX162149	MG949493	MG949676	KJ777251
Gilbertiodendron sp	Sosef 2502 (WAG)	KX162151	MG949494	MG949678	KJ777261
Gilbertiodendron sp	Wieringa 5253 (WAG)	KX162154	MG949497	MG949681	KJ777264
Gilbertiodendron splendidum	Jongkind 8782 (WAG)	KX162156	KY313270	KY312992	KJ777265
Gilbertiodendron stipulaceum	Schoenmaker 313 (WAG)	KX162157	––	MG949684	KJ777266
Gilbertiodendron stipulaceum	Schoenmaker 385 (WAG)	KX162158	––	MG949685	KJ777267
Gilbertiodendron stipulaceum	Wieringa 1644 (WAG)	KX162159	KY313271	KY312993	KJ777268
Gilbertiodendron tonkolili	Burgt 1423 (WAG)	KX162160	KY313272	KY312994	KJ777269
Gilbertiodendron tonkolili	Burgt 1457 (WAG)	KX162161	MG949500	MG949686	KJ777270
Gilbertiodendron unijugum	Burgt 73 (WAG)	KX162162	KY313273	KY312995	KJ777271
Gilbertiodendron unijugum	McPherson 15847 (WAG)	KX162163	MG949501	MG949687	KJ777272
Gilbertiodendron unijugum	Wieringa 1075 (WAG)	KX162164	MG949502	MG949688	KJ777273
Gilbertiodendron unijugum	Wilde 10974 (WAG)	KX162165	KY313274	KY312996	KJ777274
Gilbertiodendron Wieringa	Wieringa 3793 (WAG)	KX162085	MG949496	MG949637	KJ777263
Gilletiodendron glandulosum	Duvall 110	KX162166	MG949505	MG949689	MG949323
Gilletiodendron pierreanum	Wieringa 2289 (WAG)	KX162168	MG949506	MG949690	AY955809
Gilletiodendron pierreanum	Breteler 14167 (WAG)	EU361957	KY313275	KY312997	KY306578
Gilletiodendron pierreanum	Breteler 14657 (WAG)	KX162167	KY313278	KY312998	KY306579
Gilletiodendron pierreanum	Wieringa 5448 (WAG)	KX162169	MG949507	MG949691	MG949373
Goniorrhachis marginata	Lewis 1909 (K)	EU361959	KY313277	KY312999	AY955776

Goniorrhachis marginata	Bruneau sn (K)	––	KY313276	KY313000	KY306580
Guibourtia coleosperma	Luwiika 330 (MO)	EU361962	KY313279	KY313001	––
Guibourtia demeusei	Wieringa 2396 (WAG)	KX162171	KY313280	KY313002	––
Guibourtia demeusei	Breteler 13041 (WAG)	––	MG949510	MG949693	––
Guibourtia ehie	Valkenburg 3053 (WAG)	KX162173	MG949512	MG949695	––
Guibourtia ehie	Breteler 14009 (WAG)	KX162172	KY313281	KY313003	AY955801
Guibourtia ehie	Breteler 13754 (WAG)	EU361963	MG949511	MG949694	MG949302
Guibourtia hymenaeifolia	Brunner 1425 (NY)	KX162174	KY313282	KY313004	AY955802
Guibourtia pellegriniana	Breteler 14537 (WAG)	EU361965	MG949515	MG949698	––
Guibourtia pellegriniana	vanBergen 425 (WAG)	KX162175	MG949513	MG949696	––
Guibourtia tessmannii	Wieringa 6281 (WAG)	KX162177	KY313283	KY313005	––
Guibourtia tessmannii	Breteler 14865 (WAG)	KX162176	MG949514	MG949697	––
Hardwickia binata	Fogere sn (MT)	EU361967	KY313284	KY313006	AY955789
Heterostemon ellipticus	Cid 8361 (NY)	––	––	KY313007	KY306581
Heterostemon ingifolius	Redden 3194 (US)	KX162178	KY313285	KY313008	FJ817525
Heterostemon mimosoides	Schulz 10415 (US)	––	MG949516	––	FJ817527
Heterostemon mimosoides	Redden 3727 (US)	KX162179	KY313286	KY313009	FJ817528
Humboldtia brunonis	Rickson sn (OSC)	KX162180	KY313287	KY313010	KY306582
Humboldtia decurrens	Rickson sn (OSC)	KX162181	KY313288	KY313011	KY306583
Humboldtia unijuga	Rickson sn (OSC)	KX162182	KY313289	KY313012	KY306584
Humboldtia vahliana	Rickson sn (OSC)	EU361970	KY313290	KY313013	KY306585
Hylodendron gabunense	Wieringa 5858 (WAG)	KX162184	KY313292	KY313015	––
Hylodendron gabunense	Breteler 15041 (WAG)	EU361971	KY313291	KY313014	KY306586
Hylodendron gabunense	Rickson sn (OSC)	KX162183	––	MG949699	MG949351
Hymenaea courbaril	Breteler 13357 (WAG)	EU361972	KY313293	KY313016	––
Hymenaea eriogyne	Harley 19016 (K)	KX162185	KY313294	KY313017	KY306587
Hymenaea oblongifolia	Klitgaard 668 (K)	EU361973	KY313295	KY313018	––
Hymenaea strigonocarpa	Saldan 324 (US)	KX162186	––	KY313019	KY306588
Hymenaea verrucosa	Herendeen 11-XII-97-3 (US)	KX162187	KY313296	KY313020	––
Hymenostegia elegans	Wieringa 6066 (WAG)	KX162188	KY313297	KY313021	KY306589
Hymenostegia elegans	Breteler 13294 (WAG)	KX162193	KY313300	KY313024	KY306592
Hymenostegia felicis	Wieringa 2841 (WAG)	KX162190	KY313298	KY313022	KY306590
Hymenostegia felicis	Wilde 11631 (WAG)	KX162191	MG949517	MG949701	MG949383
Hymenostegia floribunda	Breteler 14334 (WAG)	KX162192	KY313299	KY313023	KY306591
Hymenostegia klainei	Wieringa 4714 (WAG)	KX162194	MG949519	––	MG949369
Hymenostegia klainei	Wieringa 5137 (WAG)	KX162196	MG949521	––	MG949372
Hymenostegia neoaubrevillei	Breteler 14793 (WAG)	KX162197	KY313301	KY313025	KY306593
Hymenostegia normandii	Wieringa 2358 (WAG)	EU361978	KY313304	KY313026	KY306594
Hymenostegia pellegrinii	Wieringa 6495 (WAG)	KX162198	KY313303	KY313027	KY306595
Hymenostegia pellegrinii	Wieringa 2581 (WAG)	KX162189	MG949518	MG949700	MG949362
Hymenostegia robusta	Wieringa 2575 (WAG)	EU361976	––	MG949702	MG949361
Hymenostegia talbotii	Wieringa 2205 (WAG)	KX162199	KY313305	KY313028	KY306596
Icuria dunensis	Jonhson & Avis 660 (WAG)	KX162201	KY313307	KY313030	––
Icuria dunensis	Dold 3362 (WAG)	EU361979	KY313306	KY313029	AF513690
Intsia bijuga	Utterridge 23 (K)	EU361981	KY313308	––	KY306597
Intsia bijuga	Wieringa 4193 (WAG)	KX162204	MG949524	––	MG949367
Intsia sp	Herendeen 28-IV-99-6 (US)	KX162203	KY313309	KY313031	KY306598
Intsia sp	Herendeen 1-V-99-2 (US)	KX162202	MG949523	MG949703	MG949329
Isoberlinia doka	Jongkind 2552 (WAG)	EU361982	KY313310	KY313032	AF513691
Isoberlinia scheffleri	Herendeen 16-XII-97-2 (US)	EU361983	MG949525	––	MG949328
Isoberlinia tomentosa	Jongkind 2560 (WAG)	KX162205	KY313311	KY313033	KY306599
Isomacrolobium explicans	Jongkind 10242 (WAG)	KX162206	KY313312	KY313034	KY306600
Isomacrolobium triplisomeris	Wieringa 4439 (WAG)	KX162208	KY313313	KY313036	KY306602
Isomacrolobium vignei	Jongkind 5684 (WAG)	––	KY313314	KY313037	KY306603
Julbernardia brieyi	Leal 186 (WAG)	KX162209	KY313315	KY313038	KY306604
Julbernardia brieyi	Wieringa 5620 (WAG)	KX162210	KY313316	KY313039	KY306605
Julbernardia brieyi	Leal 50 (WAG)	EU361985	MG949526	MG949704	MG949339
Julbernardia hochreutineri	Breteler 13009 (WAG)	KX162211	KY313317	KY313040	AF513693
Julbernardia hochreutineri	Wieringa 5128 (WAG)	KX162212	MG949527	MG949705	MG949371
Julbernardia magnistipulata	Herendeen 15-XII-97-1 (US)	KX162213	KY313318	KY313041	––

Julbernardia seretii	Rickson sn (OSC)	KX162214	KY313319	--	KY306606
Julbernardia sp	Breteler 14704 (WAG)	KX162215	MG949528	MG949706	--
Julbernardia sp	Wieringa 4079 (WAG)	KX162216	MG949529	--	MG949366
Leonardoxa africana	Wieringa 2113 (WAG)	EU361992	KY313323	KY313042	KY306607
Leonardoxa africana africana	Wieringa5863 (WAG)	KX162217	KY313321	KY313043	KY306608
Leonardoxa africana gracilicaulis	Wieringa4700 (WAG)	KX162218	KY313322	--	KY306609
Librevillea klainei	Wieringa 2554 (WAG)	EU361993	KY313325	KY313045	AF513696
Librevillea klainei	Sosef 2505 (WAG)	KX162219	KY313324	KY313044	KJ777275
Librevillea klainei	van Bergen 423 (WAG)	KX162220	MG949530	MG949707	MG949386
Loesenera gabonensis	Wieringa 5542 (WAG)	KX162026	KY313207	KY312933	KY306547
Loesenera gabonensis	Breteler 13988 (WAG)	KX162221	KY313326	--	KY306610
Loesenera kalantha	Breteler 13374 (WAG)	EU361994	KY313327	KY313046	KY306611
Loesenera talbotii	Cheek 7721 (K)	KX162222	KY313328	KY313047	KY306612
Loesenera walkeri	Wieringa 4542 (WAG)	KX162223	KY313329	KY313048	KY306613
Lysidice rhodostegia	Manos 1422 (DUKE)	EU361995	KY313330	--	KY306614
Lysidice rhodostegia	Rickson sn (OSC)	KX162224	KY313331	KY313049	KY306615
Macrolobium acaciifolium	Korning 47735	KX162225	KY313332	KY313050	KY306616
Macrolobium acaciifolium	Redden 1098 (US)	KX162226	--	MF987573	MF987607
Macrolobium archeri	Klitgaard 683 (K)	KX162227	KY313333	KY313051	KY306617
Macrolobium bifolium	Clarke 7712 (US)	KX162228	KY313334	KY313052	KY306618
Macrolobium bifolium	van Ogtrop 2 (WAG)	KX162229	MG949531	MF987574	MF987613
Macrolobium campestre	Redden 3649 (US)	KX162230	KY313335	KY313053	FJ817499
Macrolobium dessleri	McPherson 15998 (MO)	KX162231	KY313337	--	KY306619
Macrolobium gracile	Redden 3687 (US)	KX162232	KY313338	KY313054	FJ817500
Macrolobium huberianum	Redden 2197 (US)	KX162233	KY313336	KY313055	KY306620
Macrolobium ischnocalyx	Klitgaard 669 (K)	EU361997	KY313339	KY313056	KY306621
Macrolobium longiracemosum	Redden 1408 (US)	KX162239	MG949533	MF987564	MF987644
Macrolobium molle	Redden 3344 (US)	KX162234	--	KY313057	KY306622
Macrolobium montanum	Breteler 13798 (WAG)	KX162235	KY313340	--	KY306623
Macrolobium multijugum	Redden 3700 (US)	KX162236	KY313341	KY313058	FJ817502
Macrolobium punctatum	Redden 3650 (US)	KX162237	KY313342	KY313059	KY306624
Macrolobium sp	Klitgaard 663 (K)	KX162238	--	MF987586	MF987641
Macrolobium suaveolens	Redden 1637 (US)	KX162240	KY313343	KY313060	KY306625
Maniltoa browneoides	Fougere 26 (MT)	KX162241	KY313344	KY313061	--
Maniltoa gemnipara	Veldhuizen 1463 (WAG)	KX162242	KY313345	KY313062	KY306626
Maniltoa grandiflora	Rickson sn (OSC)	KX162243	MG949534	--	MG949352
Maniltoa lenticellata	Wieringa 4176 (WAG)	KX162245	KY313346	KY313063	KY306627
Maniltoa lenticellata	Gray 8937 (QRS)	KX162244	MG949535	--	MG949326
Microberlinia bisulcata	Rickson sn (OSC)	EU362002	KY313347	--	KY306628
Microberlinia brazzavillensis	Wieringa 2516 (WAG)	EU362003	KY313348	KY313064	AF513697
Microberlinia brazzavillensis	Mouandza Mbembo 91 (WAG	KX162246	MG949536	MG949708	MG949341
Neochevalierodendron stephanii	Breteler 13262 (WAG)	EU362006	KY313349	KY313065	KY306629
Normandiodendron bequaertii	Wilde 11209 (WAG)	EU362007	KY313350	KY313066	KY306630
Oddoniodendron micranthum	Breteler 14181 (WAG)	EU362008	KY313351	KY313067	KY306631
Oddoniodendron micranthum	Wieringa 6165 (WAG)	KX162247	KY313352	KY313068	KY306632
Oddoniodendron micranthum	Wieringa 6189 (WAG)	KX162248	MG949537	MG949709	MG949378
Oddoniodendron sp	Breteler 14583 (WAG)	KX162249	MG949538	MG949710	MG949307
Paloue guianensis	Lindeman 1980/874 (U)	KX162250	KY313355	--	KY306633
Paloue guianensis	Bordenave 871 (U)	KX162251	MG949542	--	MG949292
Paloue induta	Clarke 7587 (US)	EU362015	KY313356	KY313069	KY306634
Paloue riparia	Redden 1161A (US)	EU362016	KY313357	KY313070	FJ817546
Paloue riparia	Redden 3314 (US)	--	MG949541	--	FJ817548
Paramacrolobium coeruleum	Breteler 13350 (WAG)	KX162252	KY313358	KY313071	KY306635
Paramacrolobium coeruleum	Herendeen 11-XII-97-1 (US)	EU362017	KY313359	KY313072	KY306636
Paramacrolobium coeruleum	Clark 203 (K)	KX162253	--	MG949711	MG949322
Paramacrolobium coeruleum	Jongkind 10430 (WAG)	KX162254	MG949543	MG949712	MG949336
Peltogyne confertiflora	Bridgewater 793 [E]	EU362021	MG949544	MG949713	AY955798
Peltogyne floribunda	Jansen-Jacobs 2652 (U)	EU362022	KY313360	KY313073	KY306637
Peltogyne paniculata	Jansen-Jacobs 5373 (U)	KX162255	KY313361	KY313074	AY955797
Peltogyne pauciflora	Harley 16146 (K)	KX162256	KY313362	KY313075	AY955799

Peltogyne venosa	Redden 1106 (US)	KX162257	KY313363	KY313076	––
Plagiosiphon emarginatus	Wieringa 6067 (WAG)	KX162261	MG949547	MG949714	KJ777276
Plagiosiphon emarginatus	Breteler 13333 (WAG)	KX162258	KY313364	KY313077	KY306638
Plagiosiphon emarginatus	Breteler 13759 (WAG)	KX162260	MG949546	––	MG949303
Plagiosiphon emarginatus	Breteler 13073 (WAG)	KX162259	MG949545	––	MG949297
Plagiosiphon emarginatus	Breteler 15391 (WAG)	KX162270	MG949554	MG949720	MG949313
Plagiosiphon emarginatus	Wieringa 3126 (WAG)	KX162271	MG949555	MG949721	MG949365
Plagiosiphon gabonensis	Sosef 2503 (WAG)	KX162263	KY313365	KY313078	KJ777277
Plagiosiphon gabonensis	Wieringa 4400 (WAG)	KX162264	MG949549	MG949715	KJ777278
Plagiosiphon gabonensis	Wieringa 6137 (WAG)	KX162265	MG949550	MG949716	KJ777279
Plagiosiphon gabonensis	Breteler 13100 (WAG)	KX162262	MG949548	––	MG949298
Plagiosiphon longitubus	Wieringa 5864 (WAG)	KX162266	MG949551	MG949717	KJ777280
Plagiosiphon longitubus	Wieringa 5870 (WAG)	KX162267	KY313366	KY313079	KJ777281
Plagiosiphon multijugus	Andel 4044 (WAG)	KX162268	MG949552	MG949718	KJ777282
Plagiosiphon multijugus	Wieringa 3813 (WAG)	KX162269	KY313367	KY313080	KJ777283
Plagiosiphon sp	Breteler 12828 (WAG)	EU361926	MG949553	MG949719	MG949296
Plagiosiphon sp nov	Wieringa 4039 (WAG)	KX162272	MG949556	MG949722	KJ777284
Polystemonanthus dinklagei	Breteler 13395 (WAG)	EU362028	KY313368	KY313081	––
Prioria balsamifera	Wieringa 3233 (WAG)	EU361960	MG949509	MG949724	AY955787
Prioria balsamifera	Breteler 10601 (WAG)	KX162273	KY313369	KY313082	KY306639
Prioria balsamifera	Breteler 14806 (WAG)	KX162274	MG949557	MG949723	MG949311
Prioria bulcholzii	Sosef 2240 (WAG)	KX162276	KY313370	KY313083	KY306640
Prioria copaifera	Foster 927 (F)	EU362030	KY313371	KY313084	AY955778
Prioria gilbertii	Mackinder 258 (K)	KX162277	KY313353	KY313085	AY955780
Prioria mannii	Bruneau 1057 (K)	EU362010	MG949539	MG949725	AY955781
Prioria mannii	Wieringa 2011 (WAG)	KX162278	KY313372	KY313086	KY306641
Prioria msoo	Herendeen 18-XII-97-1 (US)	EU362011	KY313354	KY313087	AY955779
Prioria oxyphylla	Breteler 14088 (WAG)	EU362012	MG949540	MG949726	AY955783
Prioria oxyphylla	Breteler 14768 (WAG)	KX162279	KY313373	KY313088	KY306642
Prioria platycarpa	Smith 7549 (US)	KX162280	KY313320	KY313089	AY955784
Saraca asosa	Fougere 32 (MT)	KX162281	––	KY313090	KY306643
Saraca declinata	Manos 1417 (DUKE)	EU362033	KY313374	KY313091	––
Saraca dives	Manos 1419 (DUKE)	KX162282	MG949558	––	––
Saraca indica	Rickson sn (OSC)	––	––	MG949727	MG949350
Saraca sp	Herendeen 24-IV-99-3 (US)	KX162284	––	MG949728	––
Saraca sp	Herendeen 1-IV-99-4 (US)	KX162283	KY313375	KY313092	KY306644
Saraca thaipingensis	Wieringa 4166 (WAG)	KX162286	KY313377	KY313094	––
Saraca thaipingensis	Rickson sn (OSC)	KX162285	KY313376	KY313093	KY306645
Schotia afra	Hodgkiss 1 (BOL)	EU362037	KY313378	KY313095	AY955774
Schotia brachypetala	Bruneau sn (K)	KX162288	KY313379	KY313096	KY306646
Schotia brachypetala	Wieringa 5960 (WAG)	KX162289	––	––	KY306647
Schotia brachypetala	Archambault 36_2602 (MT)	KX162287	––	MG949729	MG949291
Scorodophloeus zenkeri	Breteler 12780 (WAG)	EU362041	KY313380	KY313097	KY306648
Scorodophloeus zenkeri	Breteler 14073 (WAG)	KX162290	KY313381	KY313098	KY306649
Sindora bruggemanii	Sosef 363 (WAG)	EU362043	KY313382	KY313099	AY955824
Sindora coriacea	Herendeen 26-IV-99-4 (US)	KX162291	MG949559	MG949730	––
Sindora coriacea	Herendeen 27-IV-99-3 (US)	KX162292	MG949560	MG949731	––
Sindora coriacea	Herendeen 29-IV-99-2 (US)	EU362044	KY313383	KY313100	KY306650
Sindora klaineana	Wieringa 5615 (WAG)	KX162293	KY313384	KY313102	––
Sindora klaineana	Breteler 14415 (WAG)	––	KY313385	KY313101	AY955819
Sindora siamensis	Herendeen 22-IV-99-1 (US)	KX162294	KY313386	KY313103	AY955821
Sindora supa	Fougere 8 (MT)	EU362047	KY313387	KY313104	AY955822
Sindora wallichii	Fougere 9 (MT)	EU362048	KY313388	KY313105	AY955820
Sindoropsis letestui	Breteler 13022 (WAG)	EU362049	KY313389	KY313106	AY955818
Stemonocoleus micranthus	Breteler 13070 (WAG)	EU362051	KY313390	KY313107	AY955795
Stemonocoleus micranthus	Dibata 1203 (WAG)	KX162295	KY313391	KY313108	KY306651
Talbotiella batesii	Wieringa 4051 (WAG)	––	KY313392	KY313109	KY306652
Talbotiella breteleri	Wieringa 5807 (WAG)	KX162296	KY313393	KY313110	KY306653
Tamarindus indica	Herendeen 29-IV-99-5 (US)	KX162297	KY313394	KY313111	KY306654
Tamarindus indica	Stevees sn (MT)	KX162298	KY313395	––	KY306655

Tamarindus indica	Velayos 11532 (MA)	KX162299	––	––	MG949357
Tessmannia africana	Breteler 12275 (WAG)	EU362057	KY313396	KY313112	KY306656
Tessmannia africana	Wieringa 5826 (WAG)	KX162300	MG949561	MG949732	MG949376
Tessmannia anomala	Breteler 14633 (WAG)	KX162301	KY313397	KY313113	AY955812
Tessmannia bambidiensis	Breteler 13201 (WAG)	KX162308	MG949567	MG949738	MG949300
Tessmannia dewildemaniana	Breteler 14618 (WAG)	KX162302	KY313398	KY313114	KY306657
Tessmannia dewildemaniana	J.J.F.E. de Wilde 11379 (WAG)	KX162303	MG949564	MG949735	MG949382
Tessmannia lescrauwaetii	Breteler 13215 (WAG)	EU362059	KY313399	KY313115	KY306658
Tessmannia lescrauwaetii	Breteler 14632 (WAG)	KX162304	MG949565	MG949736	MG949309
Tessmannia marantha	Wieringa 5476 (WAG)	KX162305	MG949566	MG949737	MG949375
Tessmannia neglecta	Breteler 12548 (WAG)	EU362058	MG949562	MG949733	AY955813
Tessmannia neglecta	Wieringa 5442 (WAG)	KX162306	KY313400	KY313116	KY306659
Tessmannia nervosa	Wieringa 3224 (WAG)	KX162307	KY313401	KY313117	KY306660
Tetraberlinia apiphylla	Wieringa 4148 (WAG)	KX162309	KY313402	KY313118	KY306661
Tetraberlinia bifoliolata	Wieringa 3304 (WAG)	EU362060	MG949569	MG949739	––
Tetraberlinia bifoliolata	Breteler 13081 (WAG)	KX162310	KY313403	KY313119	KY306662
Tetraberlinia bifoliolata	Leal 39 (WAG)	KX162311	MG949568	––	MG949338
Tetraberlinia bifoliolata	Wieringa 5097 (WAG)	KX162312	MG949570	MG949740	MG949370
Tetraberlinia moreliana	Wieringa 3156a (WAG)	KX162195	MG949520	––	––
Tetraberlinia moreliana	Breteler 13097 (WAG)	KX162313	KY313404	KY313120	AF513702
Tetraberlinia moreliana	Wieringa 2366 (WAG)	KX162315	MG949571	MG949742	AF513703
Tetraberlinia moreliana	Wieringa 3165 (WAG)	KX162316	MG949572	MG949743	AF513704
Tetraberlinia moreliana	Wieringa 2124 (WAG)	KX162314	––	MG949741	MG949358
Tetraberlinia moreliana	Wilde sn (WAG)	KX162317	MG949573	MG949744	MG949385
Tetraberlinia polyphylla	Wieringa 3151 (WAG)	EU362061	KY313405	KY313121	AF513705
Tetraberlinia polyphylla	Wieringa 3123 (WAG)	KX162318	MG949574	MG949745	AF513706
Zenia insignis	LiaoWenBo 1 (US)	KX162319	KY313406	KY313122	KY306663
Zenkerella citrina	Cheek 7614 (K)	EU362066	KY313407	KY313123	KY306664
Zenkerella egregia	Kayombo 5325 (MO)	KX162320	KY313408	KY313124	KY306665

A new phylogeny-based tribal classification of subfamily Detarioideae, an early branching clade of florally diverse tropical arborescent legumes.

Manuel de la Estrella, Félix Forest, Bente Klitgård, Gwilym P. Lewis, Barbara A. Mackinder, Luciano P. de Queiroz, Jan J. Wieringa & Anne Bruneau

Figure S1. Maximum likelihood tree for the combined alignment of ITS and plastid markers (matK, trnG-trnG2G, rpL16). Branch widths correspond to bootstrap values. Bootstrap values are presented for each node.

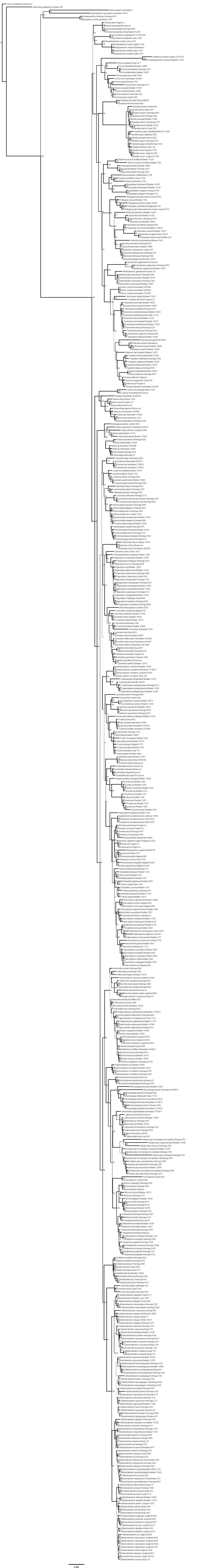

Supplement: Supplementary file 1 — Supplementary Information [file 41598_2018_24687_MOESM1_ESM.pdf]
